# Genetic Potential and Inheritance Pattern of Phenological Growth and Drought Tolerance in Cotton (*Gossypium Hirsutum* L.)

**DOI:** 10.3389/fpls.2021.705392

**Published:** 2021-09-24

**Authors:** Tahir Mahmood, Xiukang Wang, Sunny Ahmar, Muhammad Abdullah, Muhammad Shahid Iqbal, Rashid Mehmood Rana, Muhammad Yasir, Shiguftah Khalid, Talha Javed, Freddy Mora-Poblete, Jen-Tsung Chen, Muhammad Kausar Nawaz Shah, Xiongming Du

**Affiliations:** ^1^State Key Laboratory of Cotton Biology, Institute of Cotton Research, Chinese Academy of Agricultural Sciences, Anyang, China; ^2^College of Life Sciences, Yan'an University, Yan'an, China; ^3^Department of Plant Breeding and Genetics, Pir Mehar Ali Shah Arid Agriculture University, Rawalpindi, Pakistan; ^4^College of Agriculture, Fujian Agriculture and Forestry University, Fuzhou, China; ^5^Institute of Biological Sciences, Universidad de Talca, Talca, Chile; ^6^Department of Life Sciences, National University of Kaohsiung, Kaohsiung, Taiwan

**Keywords:** climate change, drought tolerance, early maturity, phenology, heritability, combining ability, inheritance

## Abstract

Cotton has prime importance in the global economy and governs socio-economic affairs of the world. Water scarcity and high temperature are major constraints that badly affect cotton production, which shows the need for the development of drought-tolerant varieties. Ten cotton genotypes, including three drought-tolerant and seven susceptible, were identified from a panel of diverse cotton genotypes at the seedling stage under two contrasting water regimes. Three lines were crossed with seven testers under line × tester mating design. The 21 F1 cross combinations along with 10 parents were evaluated under 100% non-stress (NS) and 50% drought stress (DS) filed capacity to assess the effects of drought stress and its inheritance in the next generation. All the genotypes were evaluated till the maturity stage for combining ability, heritability, and other genetic factors to understand the drought tolerance mechanisms. The proportional contribution of lines in the total variance evidenced that lines had a significant higher contribution in total variance for days to boll opening (DBO) of 10% and proline contents (PC) of 13% under DS conditions. It indicates that lines contributed more positive alleles for such traits. Under DS condition, DTV-9 × BT-252 and DTV-9 × DTV-10 had maximum negative specific combining ability (SCA) effects for DBO. Simultaneously, DBO also had higher heritability (h2) which indicates its dominant gene action and meanwhile, the importance of these combinations for the early mature and short duration variety development. The results revealed that most of the studied traits, including days taken to maturity, yield traits, and physiological traits, are under significant genetic control, with a strong genetic basis and have a huge potential for improving drought tolerance in cotton. Drought tolerance was found to have a strong association with early maturity and agro-climatic conditions of the cultivated areas. Identified superior parents in this study are suggested to use in the future breeding program to advance the cotton growth and drought tolerance.

## Introduction

**Figure 1:**
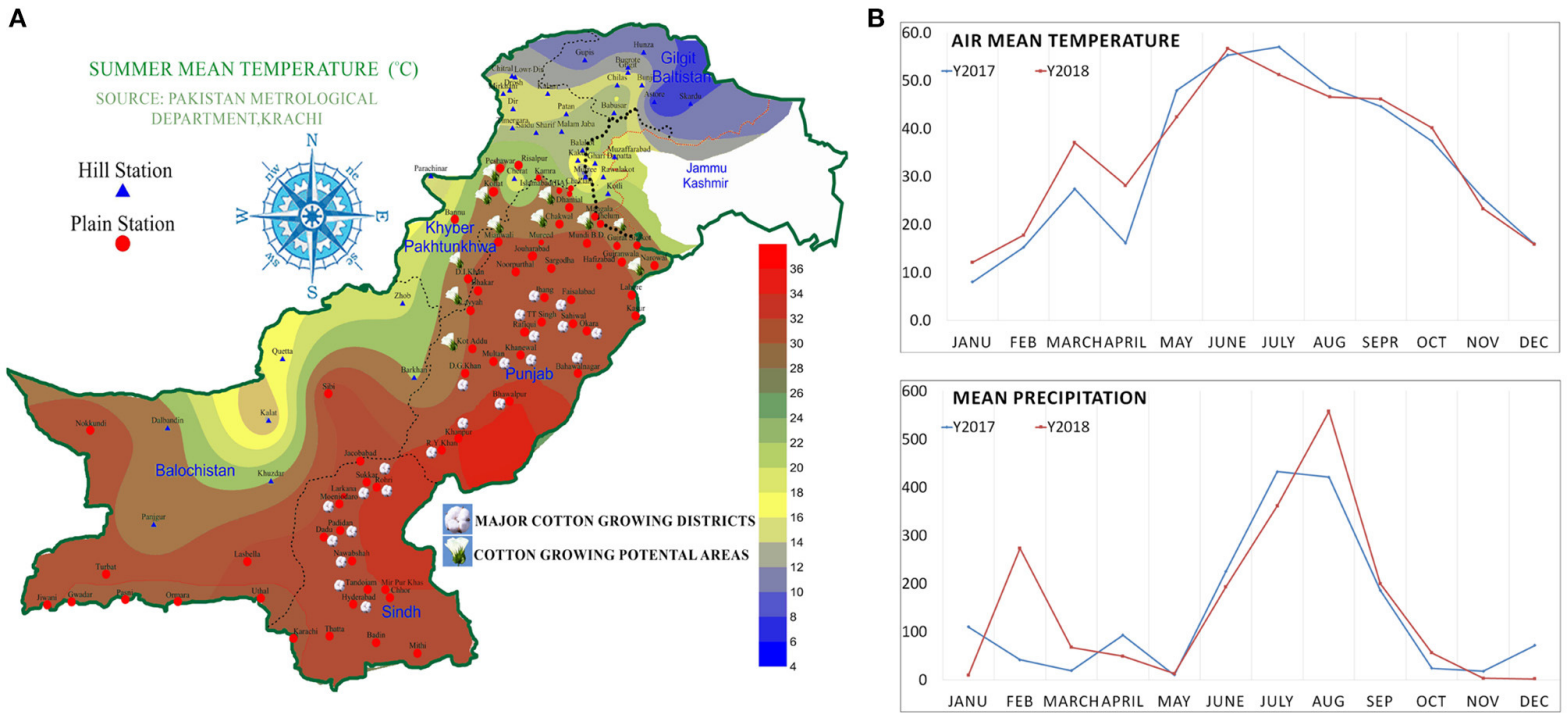
Summer mean temperature map of Pakistan, **(A)** major cotton growing and potential areas of Pakistan. **(B)** Mean air temperature and mean precipitation of Rawalpindi, Pakistan in 2017 and 2018.

Cotton is a principal fiber crop, providing 35% of the total fiber needs of the world, and is known as white gold. It is mostly grown in warmer climates. Thus, unconditionally, cotton is exposed to drought and high temperature (Abdelraheem et al., 2019). In Pakistan, mostly cotton is grown in two regions: the southern part of Punjab and some districts of Sindh (Figure 1). Cotton is considered the lifeline of the economy of Pakistan. It shares 0.8% of gross domestic production (GDP) and contributes 4.5% of agricultural value addition (Pakistan economic survey 2019-20).

During the development, growth, and reproduction stages, plants illustrate more vulnerable behavior to abiotic stresses. Drought and heat stress are the major factors that limit crop productivity and leads to a substantial reduction in final yield. Different physiological, morphological, biochemical, and molecular mechanisms have evolved in plants to overcome drought stress (Abid et al., 2016; Mahmood et al., 2020b). These mechanisms reallocate the resources away from reproduction and growth. In the case of cotton, drought and heat stress, additional inputs like more irrigation water are required to maintain growth and productivity. These management practices increase yield cost and production unsustainability in cotton. Consequently, effective strategies should be designed to overcome abiotic stress losses while improving and maintaining current yield losses (Kerr et al., 2018).

Environmental stresses cause over-production of various reactive oxygen species (ROS), which leads to oxidation of several proteins, carbohydrates, lipids and DNA, and peroxidation of cellular membranes (Zafar et al., 2018; Hussain et al., 2019). Antioxidant defense components maintain the balance between ROS production and their scavenging agents. It comprises two wings: one is non-enzymatic antioxidants including proline, phenolics, tocopherols, ascorbate, and carotenoids, and the second enzymatic components such as catalase (CAT), peroxidases (PODs), and superoxide dismutase (SOD) (Das and Roychoudhury, 2014; Wu et al., 2015). These defense components are firstly converted to active oxygen (${\text{O}}_{2}^{-}$) into nontoxic components such as H_2_O_2_, H_2_O, and O_2_. Osmotic adjustment (OA) facilitates antioxidants defense machinery by the accumulation of proline (Singh et al., 2015).

Additionally, leaf water potential causes stomatal closure, leading to CO_2_ fixation and finally reducing the photosynthetic process. Photosynthetic apparatus damaged by the disturbance of chlorophyll contents and other photosynthates (Parida et al., 2007). Drought tolerance is associated with higher proline contents and is improved relative to water content (RWC) maintenance under drought stress (Tian et al., 2019). Accumulation of these osmo-protectants endorses cell membrane stability and OA to protect the cell functions and cellular membranes in cotton (Rahman et al., 2008). Rahman et al. (2008) also reported a substantial association between the number of bolls and cottonseed yield. Significant effect of drought stress on early seedling growth, root, and shoot parameters was recently observed (Singh et al., 2018). These physiological traits ultimately contribute to plant yield and boll number, boll weight, and finally, cottonseed yield.

According to phenological and morphological perspectives, to define the earliness and short growth period is much challenging and complex. Emerging of first sympodial branch, first square and first flower are some of the earliness indicators (Bednarz and Nichols, 2005). Shorter squaring period (Hesketh and Low, 1968), early and more flowering sites per sympodial branch (Hesketh et al., 1975), early and shorter boll maturity (Gipson and Ray, 1970), fast seedling growth and vigor gain (Leffler, 1979), and seedling stress tolerance (Muramoto et al., 1971) are some earliness influencing factors in cotton. Probably, earliness and short growth period is a combination of various possible venues. Advances in early maturity is possible through modifications in the initiation of squaring, flowering intervals, boll filling periods, and plant growth period. Perhaps, if flowering interval was reduced by just one day, it, alone, could shorten the whole growth period by more than one week (Bednarz and Nichols, 2005). Early maturity and a short growth period provide a chance to escape the drought spell and minimize the yield losses.

Many conventional approaches and strategies have been adopted to improve plant drought tolerance as a natural selection of best-performing plants under stress conditions. Still, this phenotypic selection procedure is slow and un-effective against complex traits such as drought tolerance with low heritability (Abdelraheem et al., 2015). Secondary approaches as indirect selection based on secondary characteristics and correlation estimation among secondary traits and yield can provide insight into drought tolerance inheritance (Saeed et al., 2011). The third approach is based on comparison under the stress and non-stress conditions, testing drought tolerance and susceptibility in germplasm, and ranking genotypes (Abdelraheem et al., 2015). A sufficient variation and heterosis of parents are needed for any breeding program. Selection and then performance-based evaluation of parent genotypes can be estimated by measuring F1 hybrid combinations combining ability. Line × tester analysis widely adopted the method to select the parents, calculate the combing ability for hybrid development, and estimate the possible gene action (Ashokkumar et al., 2014). This design provides insight into the specific combining ability (SCA) of crosses, general combining ability (GCA) of parents, and other vital genetic parameters. The genotypes having less environmental influence are preferred for hybrid development because of their wide stability and adaptability. Single environment studies may not provide a complete and precise estimation of combining ability and gene action (Dwivedi et al., 1999). Previously, most of the genetic structure and combining ability studies were conducted under a single environment by line × tester analysis for yield and various yield components. Recently, few researchers used multi-environment to study the combining ability for different biochemical traits (Abid et al., 2016) and yield traits (Patil et al., 2018).

Keeping in mind that cotton is susceptible to abiotic factors, under the global water scarcity situation, it is imperative to explore the genetic and inheritance pattern of morpho-physiological and biochemical traits of cotton against drought stress. The major objectives of the study were (i) screening the germplasm of cotton for drought tolerance and yield, (ii) development of breeding material of cotton as a hybrid population, (iii) undertaking the genetic and physiological mechanisms and morphological traits contributing to drought tolerance under variable moisture levels, and (iv) inheritance pattern and association among morpho-physiological and biochemical traits in intraspecific crosses of cotton under variable moisture regimes. Considering the inheritance behavior of drought tolerance and the differential response of cotton genotype toward drought stress, new breeding objectives and strategies were proposed to cop the drought stress damages in cotton. These comparative physiological and phenotypic analyses can afford the dehydration resilience and evolving characteristics of cotton. New breeding objectives and strategies were also proposed to explore the non-conventional cotton cultivation practices and areas to encounter the climate shift in Pakistan and globally.

## Materials and Methods

### Plant Material

The major experiments were splinted into three sets of experiments: first, screening experiment; second, development and evaluation of breeding material; and third, field performance experiment. The first experiment consisted of 16 genetically diverse *Bacillus thuringiensis* (Bt) and non-Bt genotypes, including 3 advanced drought tolerance varieties (DTV) and 13 high-yielding varieties of cotton, which were collected from the various institutes working on cotton in Punjab, Pakistan. The second experiment consisted of 31 genotypes including 10 parents, identified from first screening experiment and 21 F1 cross combination (see section Development of Breeding Materials for more details). The third experiment consisted of 10 selected parent genotypes in the field performance experiment. All the experiments were conducted at the department research area of Plant Breeding and Genetics, at Pir Mehr Ali Shah Arid Agriculture University Rawalpindi, Pakistan. Air mean temperature and mean precipitation of Rawalpindi district is given in Figure 1. Rawalpindi district and pothohar region, generally have sandy loam and alkaline soil types (Fateh et al., 2006).

### Experimental Conditions for the Screening Experiment

A screening experiment was conducted under controlled conditions in the first half of 2016-2017. Seeds of 16 diverse genotypes were sown in polythene bags filled with the mixture of sand, silt, and farmyard manure (FYM) in a 1:1:1 ratio, at two moisture levels, 100% filed capacity (non-stress, NS), and 50% filed capacity (drought stress, DS) in a glasshouse under controlled conditions. Ten seedlings of each genotype were planted under a factorial complete randomized design (CRD). The glasshouse temperatures were controlled as 30°C ± 2 (day) and 20°C ± 2 (night) through automatic heating and cooling systems. Five weeks after the commencement of drought stress treatments, data were recorded for the morpho-physiological and biochemical traits. The traits included were relative water content (RWC), excised leaf water loss (ELWL), chlorophyll a (ChA), chlorophyll b (ChB), total chlorophyll (ChT), chlorophyll a/b (ChA/B), proline contents (PC), beta-carotenoids (BC) plant fresh weight (PFW), plant dry weight (PDW), shoot length (SL), root and shoot ratio (R/S) fresh shoot weight (FSW), dry shoot weight (DSW), root length (RL), fresh root weight (FRW), and dry root weight (DRW).

### Development of Breeding Materials

Ten genotypes for parents were selected to develop the breeding material. Selected genotypes were shelved for one cycle to ensure the parent seed purity to develop the F0 seed in the growing season of 2016-2017 (list of parents and cross combinations used in the study is given in Supplementary Table S1). The breeding block experiment was conducted to attain a vigorous parent plant for healthy F0 seed production. Three drought-tolerant lines (DTV-9, BT-992, and MNH-886) and seven drought susceptible testers (MNH-988, DTV-3, DTV-10, BT-252, BT-555, BT-666, and FH-942) were grown in five meter and tree meter long rows, respectively. Row to row (60 cm) and plant to plant (30 cm) distance was maintained. All recommended cultural practices were adopted to get the healthy crop stand and seed. Finally, according to line × tester (7 × 3) mating design, 21 cross combinations were made to produce F0 seed at the flowering stage. Here, MNH-886 was a moderate tolerant but high yielding line. All the parent genotypes were evaluated in the first experiment for drought tolerance based on morpho-physiological and biochemical parameters at the early seedling stages (see section Experimental Conditions for the Screening Experiment). The single-ruler ginning machine used to get F0 seed from the hand-picked and cross-pollinated bolls.

### Evaluation of Breeding Material

The breeding material evaluation experiment was comprised 21, F1 combinations along with their ten parents. Ten seeds of each genotype, including parents and crosses, were sown in five earthen pots per genotype and treatment. Earthen pots of 30 × 40 cm size containing 10 kg mixture of sand, silt, and F.Y.M by 1:1:1 ratio to provide the homogeneous soil media to experimental units. Five pots per treatment and replication for each genotype were intended according to a randomized complete block designed (RCBD) to control the environmental variation. Germinated seeds were counted regularly to calculate the germination percentage (GP), and 50% germination was noted to obtain the days to germination (DG). Final experiment evaluations were conducted under rain exclusion shelter (a tunnel structure covered with polythene sheet but open from both ends) at the research area of PMAS Arid Agriculture University Rawalpindi, Pakistan during the growing season of 2017-2018. After completing the germination data recording on the 15th day after sowing, plant thinning was done to maintain two healthy plants per pot, which finally held only one plant at the drought treatment application stage. Subsequently, the data of phenological traits including days to squaring (DS), days to flowering (DF), and days to boll opening (DBO) was recorded. Physiological traits data were measured at the peak flowering stage due to it being an optimum stage of biochemical and physiological components to measure drought tolerance. Various morpho-physiological traits including plant height (PH), number of bolls per plant (NB), boll weight (BW), yield of seed cotton or yield per plant (YP), proline content (PC) (Bates et al., 1973), chlorophyll a (ChA), chlorophyll b (ChB), chlorophyll ab (ChA/B) or total chlorophyll (ChT), canopy temperature (CT), and cell membrane stability (CMS) were measured.

### Exposure of Drought Stress

Two moisture levels at field capacity, non-stress (100% FC), and drought stress (50% FC) were applied to both screening (seedling experiment) and evaluating experiment (maturity). Commencement of drought stress was employed on the true leaf stage. In evaluating experiment seven, subsequent stress intervals were enforced by maintaining drought stress treatment for 10 days per interval. After 70 days of seven consequent stress cycles, data was collected for all studied traits. The field capacity of 100 g soil sample was measure through the following formula derived from the definition of field capacity by Rubens et al. (2015).


$$\begin{array}{l} \text{Field capacity}=\text{Weight of saturated soil (100 g soil)}-\\ \text{ Dry weight of soil (100 g soil)} \end{array}$$

Weight differences of fully saturated and dry soil were measured in grams, which were nearly equal to the amount of water volume required to saturate the field capacity of 100 g soil thoroughly. The field capacity of each pot was measured by this formula: 2,000 ml water for each pot having 10,000 g of soil. After the 15 days of plant emergence, two different levels, 100% field capacity (non-stress, NS), and 50% field capacity (drought stress, DS) were maintained on a gravimetric basis (Nachabe and Member, 1998). These field capacity levels were maintained up to harvesting.

### Field Performance of Selected Genotypes Under Rainfed Conditions

Field performance of 10 selected genotypes was tested under rainfed conditions considering mild drought stress. Ten genotypes were planted in the field under randomized complete block design (RCBD) with three replications. The experiment was completely reliant on rainfall except for the initial two supplement irrigations. Plant growth and crop stand were presented in the supplementary data (Supplementary Figure S2). The data of phenological traits including days to germination (DG), days to true leaf (DTL), days to first branch (DB) days to squaring (DS), days to flowering (DF), and days to boll opening (DBO) was recorded. The data of physiological traits data were measured at flowering stage. Various morpho-physiological traits including plant height (PH), number of bolls per plant (NB), boll weight (BW), yield of seed cotton or yield per plant (YP), proline content (PC) (Bates et al., 1973), chlorophyll contents (ChC, SPAD 502 plus), leaf area (LA), leaf fresh weight (LFW), leaf dry weight (LDW), leaf water contents (LWC), leaf solutes (LS), osmolality (OS), and canopy temperature (CT) were measured.

### Measurements of Other Physiological and Morphological Parameters

Measurement of remaining morphological traits (seedling traits including RFW, RDW, SFW, SDW, PTFW, PTDW, SL, RL R/S, and maturity traits including PH, NB, BW, YP) was done manually with measuring tape and electric weighing balance. Measurements of physiological traits like excise leaf water content (ELWC) (Clarke and Mccaig, 1982), relative water content (RWC) by Clarke and Townley-Smith (1986), proline content (Bates et al., 1973). Changes in the enzymatic and non-enzymatic activity (H_2_O_2_, SOD, NOX, POX, CAT, GR, and APX) were measured (Tang et al., 2010; Zhang et al., 2012). To calculate the chlorophyll contents, including Chlorophyll a, b, and total chlorophyll contents, the equations proposed by Nagata and Yamashita (1992) were used.


$$\begin{array}{l} \text{Chlorophyll a (mg/100 ml)}=\text{0.999A663 − .0989A645} \end{array}$$

$$\begin{array}{l} \text{Chlorophyll b (mg/100 ml)}=\text{−0.328A663 + 1.77A645} \end{array}$$

(A453, A505, A645, and A663 are absorbance at 453, 505, 645, and 663 nm, respectively).

Cell membrane stability was calculated by the total electrolyte leakage measures from the leaves of stressed and typical plants (Blum and Ebercon, 1981) by the following formula below. Recently, it has been used in cotton (Saleem et al., 2015) and also in other crops such as wheat (Naeem et al., 2016) to measure the stress tolerance.


$$\text{CMS\%}=[(1-({\text{T}}_{1}/{\text{T}}_{2}))/(1-({\text{C}}_{1}/{\text{C}}_{2}))]\;\text{x}100$$

Here,

- T1 = EC of the sap of discs from the stressed plant, before autoclaving.
- T2 = EC of the sap of discs from the stressed plant, after autoclaving.
- C1 = EC of the sap of discs from the normal plant, before autoclaving.
- C2 = EC of the sap of discs from the normal plant, after autoclaving.

### Data Analyses

In the pre-screening and seedling experiment, drought stress responses (DSRI) and cumulative drought stress response index (CDSRI) were calculated based on the mean and standard deviation values of all studied traits (Awasthi et al., 2018). The genotypes were sorted and classified into three groups based on CDSRI with a difference of 3.5 (CDSRI_Maximum_ - CDSRI_Minimum_/3) bases.

❖ DSRI (individual trait) = value under DS/value under NS
❖ CDSRI = DSRI_1 + DSRI_2+… DSRI-nth

**Figure 2:**
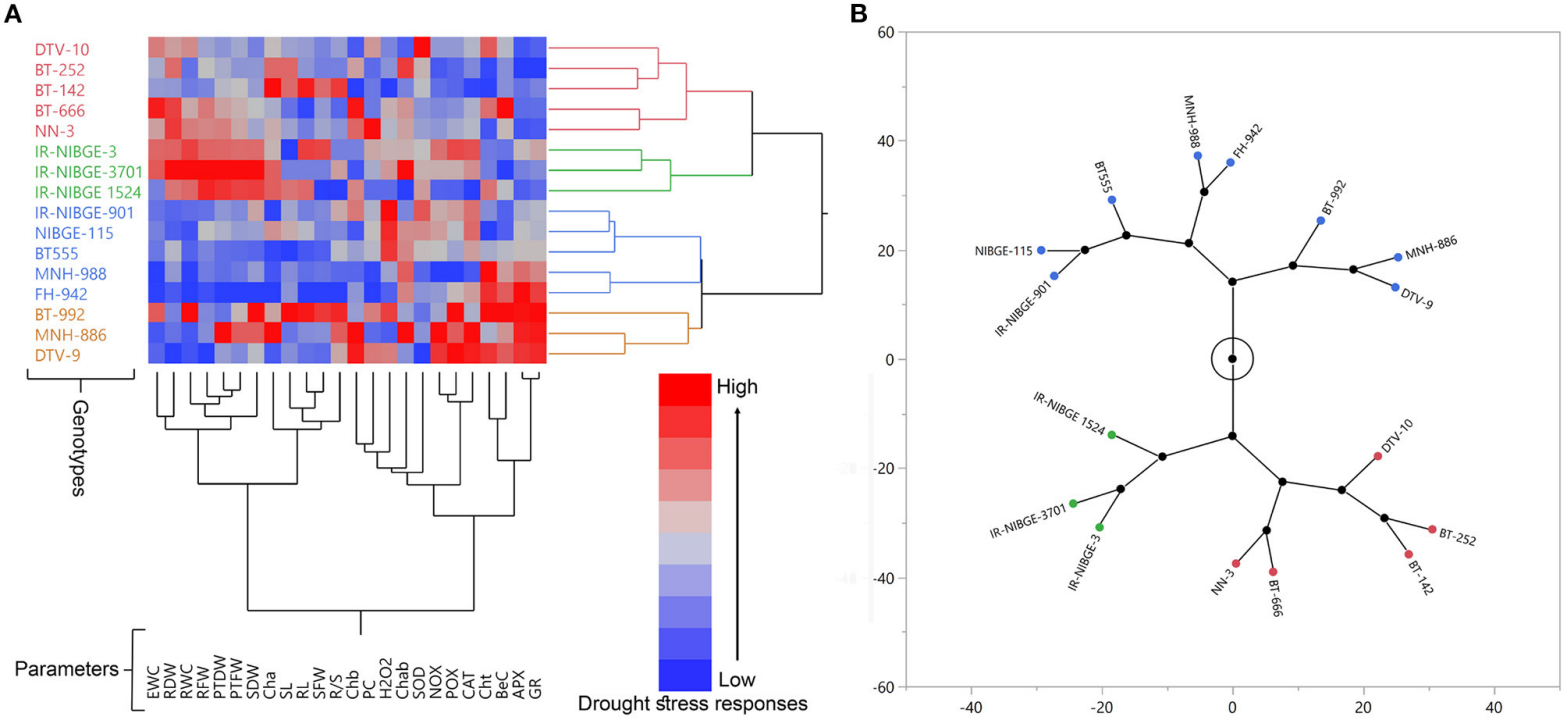
Drought stress responses and variation among a panel of diverse 16 cotton genotypes. Hierarchical Cluster and Dendrogram on the bases of seedling traits **(A)**, and Constellation Plot **(B)**. Genotypes are distributed in two major and four minor groups. In contrast, evaluated parameters are distributed in two major and five minor groups **(A)**.

**Figure 3:**
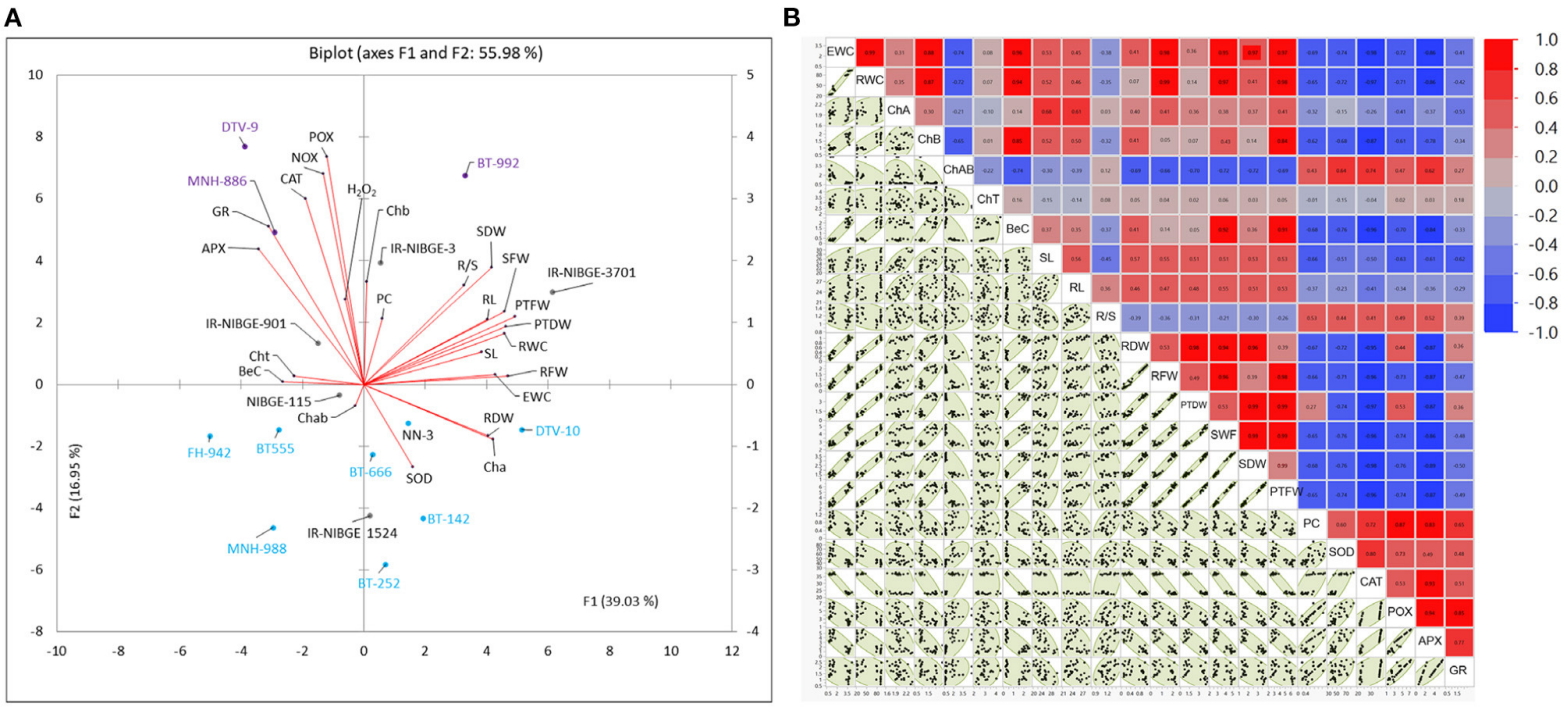
Biplot (principal component analysis) **(A)** and correlation matrix plot **(B)** for drought stress response indices of physiological, biochemical, and seedling traits under non-stress (NS) and drought stress (DS) conditions. Scatterplot showing drought tolerant (purple), moderate (orange), and susceptible (blue) cotton genotypes. Genotypes in purple color have been identified as drought-tolerant genotypes, while in blue, they have been identified as susceptible genotypes.

Drought stress response index for individual traits was calculated. The values are then summed-up to calculate one value of CDSRI for each genotype according to Awasthi et al. (2018). The DRI was used to construct clusters and constellation plots (Figure 2). Biplot analysis was performed through XLSTAT (Version 2016) to estimate the variation and association among studied traits while JMP® [Version (15.0), SAS Institute Inc., Cary, NC, USA, 1989–2019] was used to do principal component analysis (PCA) and other diversity analyses (plots and graphs in Figures 2, 3). To estimate the genetic and variance components, Residual or Restricted Maximum Likelihood (REML) balanced L × T (Line × tester) and combining ability analysis was performed for multi-environment RCBD (randomized complete block design) through AGD-R [2015 Centro Internacional de Mejoramiento de Maíz y Trigo (CIMMYT)]. The ratio of ϭ^2^GCA/ϭ ^2^SCA was used to determine the predominance of gene action (additive or non-additive) in the expression of the characters was determined, and the degree of dominance was computed by using the following formula (Kargbo et al., 2019).


$$\text{Degree of dominance = Sqrt}\;({ϭ}^{\text{2}}\text{H/}{ϭ}^{\text{2}}\text{D})$$

## Results

### Screening of Material and Selection of Parents

Out of 16, 10 genotypes for parents were selected to develop the breeding material, based on seedling and physiological traits under two different water regimes: non-stressed (NS) and drought-stressed (DS). A significant variation was present in the panel of 16 cotton genotypes for all studied parameters, with some exceptions under the DS condition. Drought stress responses (DSRI) was calculated and used to construct hierarchical cluster, dendrogram, and constellation plot (Figure 2). CDSRI was calculated based on mean and standard deviation values of all studied traits. The genotypes were sorted and classified into three groups based on CDSRI with a difference of 3.5 (CDSRI_Maximum_ - CDSRI_Minimum_/3) CDSRI (Supplementary Table S2) according to Mahmood et al. (2020a). Five genotypes, including DTV-9, BT-992, NN-3, MNH-886, and IR-NIBGE-3, were identified as drought-tolerant having higher CDSRI ranging from (32.44 to 36.16). Drought susceptible genotypes including FH-942, IR-NIBGE 1524, BT-666, MNH-988, DTV-10, BT-252, and BT-142 were secured with less CDSRI (25.07–29.11). The remaining four genotypes, including IR-NIBGE-3701, NIBGE-115, IR-NIBGE-901, and BT555, were counted as moderate tolerant genotypes.

### Multivariate, Principal Component, and Biplot Analysis

Principal component and genotype by trait biplot (GTB), a multivariate analysis was conducted to investigate the response and variability of 16 cotton genotypes for drought stress tolerance. Recently, Mahmood et al. (2020a) used the same approach to explain the variation and distribution of genotypes to words with different stress levels. Variability of drought tolerance indices was explained for the PCA1 (F1) and PCA2 (F2). Biplot demonstrating overall variability of 55.89% explained for all traits (Figure 3). Vector magnitude of biochemical traits clarified more relative, indicating the importance of these traits for selecting cotton genotypes at early seedling stages under drought stress. Distribution and position of drought-tolerant genotypes including DTV-9, BT-992, and MNH-886 fall near to the vectors of biochemical parameters including PC, POX, NOX, APX, H_2_O_2_, and SOD, which indicated the response of these genotypes for biochemical parameters. Comparatively, selected, drought susceptible genotypes including FH-942, BT-666, MNH-988, DTV-10, BT-252, and BT-142 fall away from the vectors of biochemical and physiological parameters.

### Parental General Combining Ability (GCA) Effects

To explore the inheritance of drought tolerance in cotton, line × tester and combining ability analysis was performed. ANOVA displayed a significant variability in 21 crosses including parents for all studied traits with some exceptions (Tables 1, 2).

**Table 1:** Mean square values (MS) of Line × Tester and combining ability (CA) analysis for all studied traits in 21 crosses including parents under NS conditions.

**SOURCE**	**DF**	**GP**	**DG**	**DS**	**DF**	**DBO**	**PH**	**NB**	**BW**	**YP**	**PC**	**ChA**	**ChB**	**ChAB**	**ChT**	**CTe**	**CMS**
REPLN	2	246.24	0.42	1.43	0.10	4.00	1.39	2.24	0.11	94.79	0.0002	0.14	0.28	1.33	0.82	0.0002	33.35
GENO	30	622.44^**^	2.02^*^	9.51^**^	12.67ns	40.68ns	192.11ns	7.23ns	1.01ns	325.26ns	0.0273ns	0.48ns	1.50ns	2.29^**^	3.26ns	0.0273ns	1095.07ns
CROSS	20	561.59^**^	0.88ns	11.39^**^	13.05ns	43.90ns	104.12ns	5.78ns	0.85ns	233.12ns	0.0320ns	0.40ns	1.19ns	1.50ns	2.50ns	0.0320ns	1211.04ns
LINE(c)	2	725.40ns	1.25ns	47.57^*^	53.48^*^	307.87^*^	60.05^*^	6.05ns	1.45^*^	508.49^*^	0.0406^*^	0.90ns	4.03^*^	2.76ns	8.72^*^	0.0406^*^	431.04ns
TEST(c)	6	260.85ns	0.96ns	5.36nsn	10.77^*^	21.84^*^	65.62^**^	6.18^**^	0.77^**^	267.92^**^	0.0298ns	0.29^*^	1.17^**^	0.99ns	2.13^**^	0.0298ns	2731.44ns
LXT (c)	12	684.66^**^	0.77ns	8.37^*^	7.46^**^	10.93^*^	130.71ns	5.53^**^	0.79ns	169.83^**^	0.0317ns	0.36^**^	0.72^**^	1.54ns	1.65^**^	0.0317ns	580.84ns
PARENT	9	474.44^*^	2.00ns	4.39s	11.56^**^	27.24^**^	351.56ns	9.87ns	1.21ns	464.00ns	0.0197ns	0.72^**^	2.10ns	4.13^**^	5.00ns	0.0197ns	957.53ns
LINE(p)	2	300.00ns	0.78ns	3.44ns	2.11ns	24.78ns	261.44^*^	3.11ns	0.26ns	65.71ns	0.0160ns	0.12ns	0.07ns	0.04ns	0.15ns	0.0160ns	956.70^*^
TEST(p)	6	438.10^*^	1.05ns	4.41ns	15.33^**^	29.75^*^	307.21ns	12.65^**^	1.73^**^	665.50^**^	0.0214^**^	0.76^**^	2.09^**^	5.31^*^	5.06^**^	0.0214^**^	1083.11^**^
L vs. T	1	1041.43ns	10.16ns	6.10ns	7.78ns	17.17ns	797.91ns	6.71ns	0.01ns	51.53ns	0.0164ns	1.70ns	6.22ns	5.24ns	14.36ns	0.0164ns	205.66ns
Cro vs. PAR	1	3171.37ns	25.09ns	18.07ns	14.93ns	97.23ns	516.81ns	12.55ns	2.36ns	919.28ns	0.0028ns	0.01ns	2.30ns	1.45ns	2.67ns	0.0028ns	13.67ns
ERROR	60	4707.53	0.92	2.85	2.01	4.38	6.15	0.67	0.06	20.60	0.0006	0.07	0.12	0.88	0.22	0.0006	51.17
**Combining ability**															
REPLN	2	192.06	0.59	3.76	0.90	1.54	4.62	2.33	0.11	69.98	0.0004	0.211	0.35	1.06	1.10	0.0004	14.68
CROSS	20	561.59ns	0.88ns	11.39^**^	13.05	43.90ns	104.12ns	5.78ns	0.85ns	233.12ns	0.0320ns	0.397^**^	1.19ns	1.50ns	2.50ns	0.032ns	1211.04ns
LINE(c)	2	725.40ns	1.25ns	47.57^*^	53.48^*^	307.87^*^	60.05ns	6.05ns	1.45^*^	508.49^*^	0.0406^*^	0.903	4.03^*^	2.76ns	8.72^*^	0.0406^*^	431.04ns
TEST(c)	6	260.85ns	0.96ns	5.36ns	10.77^**^	21.84^*^	65.62^**^	6.18^**^	0.77^**^	267.92^**^	0.0298ns	0.293^**^	1.17^*^	0.99ns	2.13^*^	0.0298ns	2731.44ns
LXT (c)	12	684.66^**^	0.77ns	8.37^*^	7.46^**^	10.93ns	130.71ns	5.53^**^	0.79ns	169.83ns	0.0317ns	0.365ns	0.72^**^	1.54ns	1.65^**^	0.0317ns	580.84ns
ERROR	40	83.73	0.70	2.66	1.92	5.02	5.40	0.72	0.05	17.62	0.0005	0.096	0.17	1.14	0.31	0.0005	59.20
CV (%)		14.11	20.93	4.46	2.56	2.31	4.1	7	5.97	9.67	11.1900	14.440	25.34	52.99	14.72	11.19	12.90

* *Significant at p < 0.05*;

** *significant at p < 0.01. BTN. G%, germination percentage; DG, days to germination; DS, days to squaring; DF, days to flowering; DB, days to boll opening; PH, plant height (cm); NB, number of bolls per plant; BW, boll weight (g); YP, yield of seed cotton/ yield per plant (g); PC, proline content; ChA, chlorophyll a; ChB, chlorophyll b; ChT, chlorophyll ab or total chlorophyll; ChAB, chlorophyll a/b;CTe, canopy temperature; CMS, cell membrane stability of 31 cotton genotypes including 10 parents and 21 cross. ns, non-sifnificant*.

**Table 2:** Mean square values (MS) of Line × Tester and combining ability (CA) analysis for all studied traits in 21 crosses including parents under DS conditions.

**SOURCE**	**DF**	**GP**	**DG**	**DS**	**DF**	**DBO**	**PH**	**NB**	**BW**	**YP**	**PC**	**ChA**	**ChB**	**ChAB**	**ChT**	**CTe**	**CMS**
REPLN	2	288.2ns	0.52ns	4.66	7.65	2.45	5.17	0.23	0.11	12.95	0.00	0.01	0.05	0.07	0.07	0.00	33.3
GENO	30	632.85ns	2.18^**^	8.95ns	12.73^**^	63.88^*^	33.95ns	2.63^*^	0.50^*^	55.27^*^	0.19^*^	0.22ns	1.48	0.93ns	2.49^*^	0.19ns	1095.1ns
CROSS	20	688.2ns	1.18ns	7.24^**^	10.35^**^	63.22^**^	32.53ns	2.32^**^	0.39ns	38.43^**^	0.22ns	0.12ns	0.85	0.89ns	1.37^*^	0.22ns	1211.0ns
LINE(c)	2	1963.5^*^	2.78ns	15.73ns	33.16ns	246.87^*^	172.71^*^	5.35ns	0.09^*^	103.16ns	1.02^**^	0.19^*^	0.26	0.07ns	0.69^*^	1.02^**^	431.0ns
TEST(c)	6	353.4^*^	1.11ns	11.22^**^	14.14^*^	80.42^**^	25.56^*^	1.51ns	0.93^**^	33.70ns	0.10^**^	0.09^**^	0.76	0.84^**^	1.13ns	0.10^**^	2731.4ns
LXT (c)	12	643.1^**^	0.94ns	3.84^*^	4.66ns	24.00^**^	12.66ns	2.22^*^	0.17^*^	30.01^*^	0.14ns	0.13ns	0.99	1.06ns	1.60ns	0.14ns	580.8ns
PARENT	9	425.5^**^	2.58^*^	12.46^**^	19.17^**^	58.89^**^	40.83^**^	2.53^*^	0.56^*^	98.71^**^	0.10^*^	0.45ns	2.33	0.73ns	4.47^*^	0.10ns	957.5^*^
LINE(p)	2	277.8ns	2.33ns	16.44ns	16.44ns	1.78ns	24.11ns	0.78ns	0.11^**^	0.12ns	0.08ns	0.33^*^	1.97^**^	1.25^*^	3.65^**^	0.08^*^	956.7^*^
TEST(p)	6	363.4^*^	0.65ns	8.87^*^	13.11^*^	36.76^*^	38.22^*^	1.76ns	0.56ns	78.34^**^	0.10^**^	0.50ns	1.88	0.21^*^	3.93ns	0.10^**^	1083.1^**^
L vs T	1	1093.5ns	14.63ns	26.01ns	60.98ns	305.91ns	89.91ns	10.67ns	1.48ns	418.14ns	0.14ns	0.45ns	5.72^*^	2.85ns	9.37^*^	0.14ns	205.7ns
Cr v PAR	1	1389.9ns	18.56ns	11.60ns	2.30ns	121.91ns	0.36ns	9.73ns	2.22ns	1.01ns	0.53ns	0.01ns	6.61^*^	3.34ns	7.11^*^	0.53ns	13.7ns
ERROR	60	75.95	0.72	1.57	3.56	6.68	6.42	0.67	0.06	10.46	0.01	0.01	0.02	0.04	0.03	0.01	51.2
**Combining ability**															
REPLN	2	220.6	0.30	1.16	4.11	5.78	1.86	0.78	0.00	7.96	0.01	0.01	0.04	0.07	0.05	0.01	14.7
CROSS	20	688.3ns	1.18^*^	7.24^**^	10.35^**^	63.22^*^	32.53ns	2.32^**^	0.39^*^	38.43^**^	0.22^*^	0.12ns	0.85ns	0.89sn	1.37^*^	0.22ns	1211.0ns
LINE(c)	2	1963.5^*^	2.78ns	15.73ns	33.16^*^	246.87^*^	172.71^*^	5.35ns	0.09ns	103.16	1.02^**^	0.19^*^	0.26^*^	0.07ns	0.69^*^	1.02^*^	431.0^*^
TEST(c)	6	353.4^*^	1.11ns	11.22^*^	14.14^*^	80.42^**^	25.56^*^	1.51ns	0.93^**^	33.70^*^	0.10^**^	0.09^**^	0.76ns	0.84^**^	1.13^**^	0.10^**^	2731.4ns
LXT (c)	12	643.1^**^	0.94ns	3.84^*^	4.66ns	24.00^*^	12.66^*^	2.22^*^	0.17^**^	30.01^*^	0.14ns	0.13ns	0.99ns	1.06ns	1.60ns	0.14ns	580.8^*^
ERROR	40	72.3	0.63	1.66	2.33	7.18	4.04	0.71	0.04	10.23	0.01	0.01	0.02	0.05	0.03	0.01	59.2
CV (%)		14.9	17.80	3.08	3.32	2.98	6.66	10.15	7.42	12.38	12.42	3.88	8.53	13.36	4.74	12.42	12.9

* *Significant at p < 0.05*;

** *significant at p < 0.01. BTN. G%, germination percentage; DG, days to germination; DS, days to squaring; DF, days to flowering; DB, days to boll opening; PH, plant height (cm); NB, number of bolls per plant; BW, boll weight (g); YP, yield of seed cotton/ yield per plant (g); PC, proline content; ChA, chlorophyll a; ChB, chlorophyll b; ChT, chlorophyll ab or total chlorophyll; ChAB, chlorophyll a/b;CTe, canopy temperature; CMS, cell membrane stability of 31 cotton genotypes including 10 parents and 21 crosses. ns, non-sifnificant*.

In the results, both line and tester parents displayed considerable variation in GCA effects across the environmental conditions (Supplementary Tables S3, S4). In terms of lines, DTV-9 had the highest significant positive GCA effects (11.11 and 6.51) for GP under both DS and NS environmental conditions, respectively. The DTV-9 had significant GCA effects for a maximum of 14 and 11 traits under both DS and NS environmental conditions, respectively. Additionally, it had a maximum negative GCA (−5.15) for YP under the NS conditions whereas BT-992 had significant GCA effects for minimum of seven and six traits under both environmental conditions DS and NS, respectively. A maximum negative GCA effect (−6.51) was observed in MNH-886 under DS conditions. Moreover, it also had the full negative GCA effect (−4.92) for GP under DS condition.

In terms of testers, the highest GCA effects (25.04) were observed in MNH-988 for SMS under both DS and NS environmental conditions while lowest and adverse GCA effects (−26.56) were observed in BT-252 for CMS under both DS and NS ecological conditions. The DTV-3 and MNH-886 had significant and higher GCA effects (4.68 and 2.59) for DBO under DS condition. The BT-666 and MNH-886 had significant and higher GCA effects (0.60 and 0.54) for BN under DS condition. MNH-988 had significant GCA effects for a maximum of 10 traits under NS environmental conditions. FH-942 had significant GCA effects for 14 traits under DS conditional conditions. DTV-10 had significant GCA effects for seven and six traits under both DS and NS environmental conditions, respectively.

GCA effects for traits and significant GCA effects in all three lines and six testers were observed for DBO. In two lines and all seven testers for ChT, the highest number of parents had significant GCA effects for these two traits under DS condition. PC had a remarkable significance of GCA effects among all the parents under both environments. DG and GP had less significant GCA effects among all parents. DBO had significant GCA effects in nine parents under DS and in just three parents under NS conditions, which was the maximum differential significance of GCA effects among all the traits. The ratio had significant GCA effects in three parents under DS, while zero parents under NS condition. DBO, BW, PC, ChA ChB, ChAB, ChT, and CMS had more differential significant GCA effects among all the traits and parents under the DS condition compared to NS condition.

### Specific Combining Ability (SCA) Effects of Crosses Combinations

Statistical results displayed remarkable variation in the SCA effects across the 21 cross combinations (Supplementary Tables S5, S6). Under NS condition, BT-992 × BT-252 and BT-992 × BT-555 significantly had the highest positive and lowest negative SCA effects of 3.56 and −2.78, respectively, for DBO. DTV-9 × BT-666 and DTV-9 × DTV-10 had significantly increased positive and lowest negative SCA effects of 10.6 and −8.17, respectively, for BW. BT-992 × DTV-3 and MNH-886 × DTV-3 had significantly highest positive and lowest negative SCA effects of 11.20 and −12.15 for YP. PH, BN, BW, YP, CTe, and CMS had more significant SCA effects among all the traits under the NS condition, indicating that breeding for agronomical traits is more feasible under non-stress conditions.

Under the DS condition, DTV-9 × MNH-988 and BT-992 × BT-666 had significantly highest positive (3.22) and lowest negative (−4.30) SCA effects for DBO. BT-992 × BT-666 and DTV-9 × BT-666 had significantly highest positive (0.29) and lowest negative (−0.31) SCA effects for BW. DTV-9 × BT-555 and MNH-886 × BT-252 had significantly highest positive (1.98) and lowest negative (−0.33) SCA effects for PC. BT-992 × FH-942 and DTV-9 × BT-666 had significantly highest positive (0.94) and lowest negative (−1.0) SCA effects for ChT. BT-992 × DTV-10 and BT-992 × DTV-3 had significantly highest positive (5.55) and lowest negative (−14.19) SCA effects for CMS. GP, DBO, BW, PC, ChA ChB, ChAB, ChT, and CMS had more significant SCA effects among all the traits under DS condition.

The DS condition, GP, DS, DBO, PC, ChA ChB, ChAB, ChT, and CMS had more significant SCA effects. DBO had significant positive and negative SCA in five and four crosses, respectively. PC had significant positive and negative SCA in six and seven crosses, respectively. ChB had significant positive and negative SCA in eight and nine crosses, respectively. ChT had significant positive and negative SCA in 8 crosses, respectively, the highest among all the traits.

### Genetic Variance Components, Contribution of Genotypes Toward Total Variance, and Heritability Estimates

**Figure 4:**
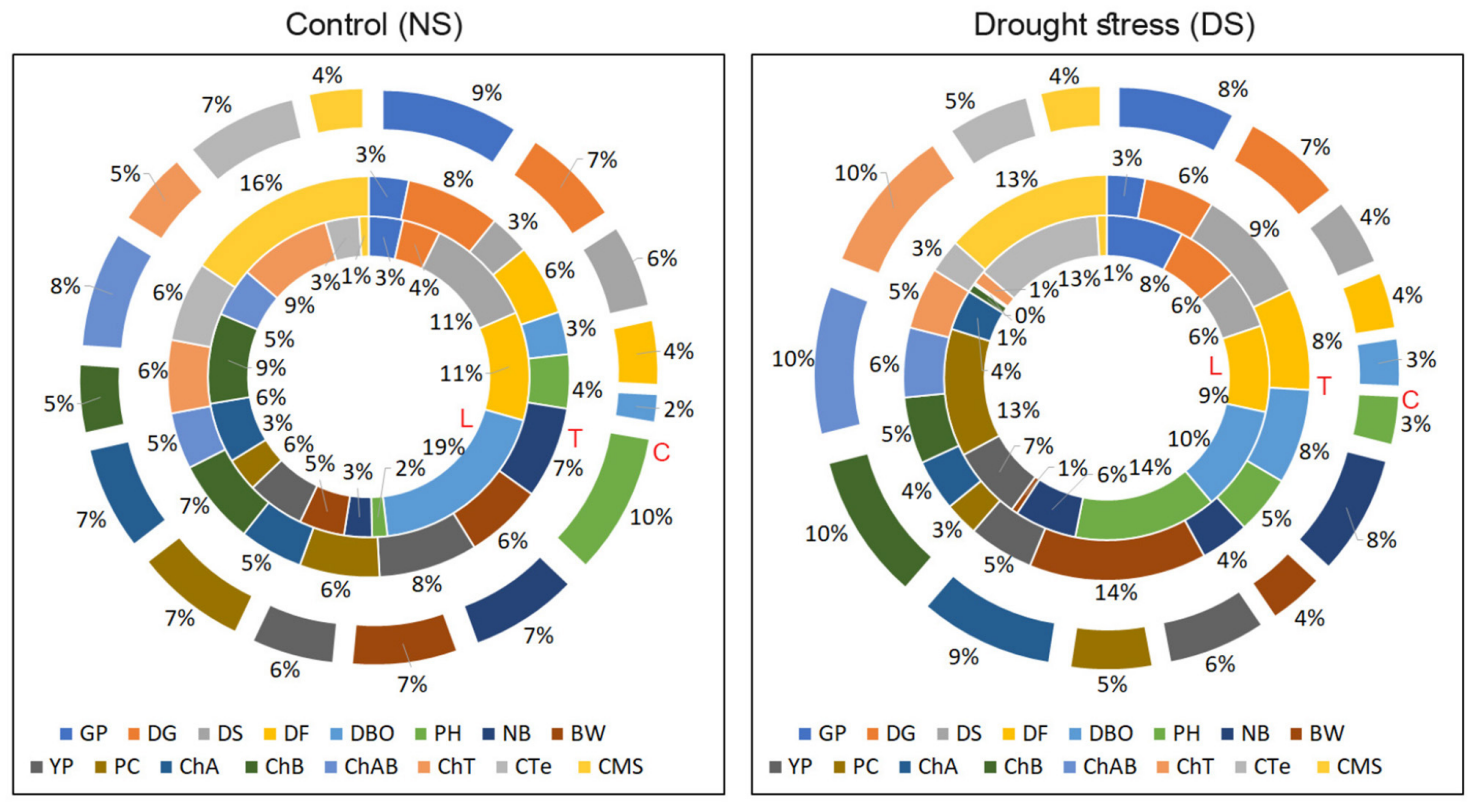
Contribution of genotypes toward total variance for studied traits under NS and DS environments (Values are given in Supplementary Table S7). L, the contribution of lines; T, the contribution of testers; C, the contribution of cross combinations.

Broad sense heritability (H^2^) and narrow-sense heritability (h^2^) displayed remarkable variation under cross environments. Germination percentage (GP) had a minimum H^2^ (0.901) and ChT had a maximum (0.998) among all the traits. In terms of h^2^, DS, BW, and PH had higher values (0.78, 0.72, and 0.722), respectively (Figure 4). The negative variance of general combining ability (σ^2^ GCA) of −0.006 ChAB was observed. A higher variance of specific combining ability (σ^2^ SCA) than σ^2^GCA was noticed for all the traits. ChA, and NB had a higher degree of dominance of 1.00, 0.98, respectively (Table 3).

**Table 3:** Estimates of genetic variance components NS and DS environments.

**NS**	**GP**	**DG**	**DS**	**DF**	**DBO**	**PH**	**NB**	**BW**	**YP**	**PC**	**ChT**	**ChA**	**ChB**	**ChAB**	**CTe**	**CMS**
σ^2^_L_	50.803	0.380	1.040	0.560	8.219	0.000	0.114	0.017	11.169	0.000	0.121	0.017	0.042	0.000	0.000	0.000
σ^2^_T_	0.000	0.000	0.000	0.000	0.000	19.020	0.249	0.077	29.499	0.000	0.047	0.000	0.044	0.042	0.000	75.717
σ^2^_LxT_	131.732	0.000	1.241	3.060	4.950	45.744	1.862	0.234	64.677	0.009	0.867	0.123	0.383	0.429	0.009	278.623
σ^2^_Gen_.	181.326	0.367	2.219	3.553	12.100	61.985	2.187	0.318	101.552	0.009	1.012	0.137	0.458	0.466	0.009	347.946
σ^2^_A_	725.305	1.470	8.875	14.211	48.401	247.938	8.747	1.272	406.208	0.036	4.048	0.549	1.830	1.866	0.036	1391.783
σ^2^_D_	526.929	0.000	4.965	12.241	19.801	182.977	7.447	0.936	258.710	0.036	3.467	0.490	1.533	1.714	0.036	1114.490
$\sigma_{\text{G}}^{2}$	1252.234	1.470	13.839	26.452	68.202	430.915	16.194	2.208	664.917	0.072	7.514	1.039	3.363	3.580	0.072	2506.273
σ^2^_E_	26.153	0.303	0.951	0.669	1.459	2.051	0.223	0.019	6.865	0.000	0.075	0.023	0.041	0.295	0.000	17.058
σ^2^_P_	1278.387	1.772	14.790	27.121	69.661	432.967	16.417	2.227	671.782	0.072	7.589	1.062	3.405	3.875	0.072	2523.331
σ^2^_GCA_	0.858	0.006	−0.692	0.001	1.648	0.002	0.145	16.411	−3.204	0.078	0.001	0.000	−0.001	0.022	0.000	0.012
σ^2^_SCA_	1.969	1.604	41.771	0.248	50.737	0.023	1.845	173.882	200.309	1.902	0.090	0.010	0.133	0.447	0.010	0.181
GA	0.436	0.004	−0.017	0.004	0.032	0.088	0.079	0.094	−0.016	0.041	0.009	0.000	−0.008	0.049	0.000	0.066
RD	0.726	0.000	0.559	0.861	0.409	0.738	0.851	0.736	0.637	1.000	0.856	0.893	0.838	0.919	1.000	0.801
G _Adv._	1276.536	2.586	15.619	25.011	85.187	436.371	15.395	2.238	714.926	0.063	7.124	0.966	3.221	3.284	0.063	2449.537
H^2^	0.980	0.829	0.936	0.975	0.979	0.995	0.986	0.992	0.990	0.997	0.990	0.978	0.988	0.924	0.997	0.993
h^2^	0.567	0.829	0.600	0.524	0.695	0.573	0.533	0.571	0.605	0.499	0.533	0.517	0.537	0.482	0.499	0.552
**DS**	**GP**	**DG**	**DS**	**DF**	**DBO**	**PH**	**NB**	**BW**	**YP**	**PC**	**ChT**	**ChA**	**ChB**	**ChAB**	**CTe**	**CMS**
σ^2^_L_	72.808	0.444	0.555	0.731	8.756	6.040	0.007	0.037	2.447	0.019	0.153	0.000	0.120	0.049	0.019	0.000
σ^2^_T_	0.000	0.000	1.016	1.167	4.053	2.071	0.006	0.073	5.679	0.000	0.000	0.000	0.010	0.000	0.000	75.717
σ^2^_LxT_	116.273	0.075	1.056	1.407	7.654	3.007	0.642	0.051	8.125	0.043	0.687	0.071	0.373	0.251	0.043	278.623
σ^2^_Gen_.	s185.627	0.487	2.462	3.058	19.063	9.178	0.654	0.149	14.937	0.062	0.820	0.071	0.489	0.295	0.062	347.946
σ^2^_A_	742.509	1.948	9.849	12.232	76.254	36.711	2.615	0.595	59.747	0.247	3.281	0.283	1.956	1.181	0.247	1391.783
σ^2^_D_	465.093	0.300	4.223	5.626	30.614	12.027	2.568	0.205	32.500	0.172	2.747	0.283	1.492	1.005	0.172	1114.490
$\sigma_{\text{G}}^{2}$	1207.602	2.248	14.073	17.859	106.868	48.738	5.183	0.799	92.248	0.419	6.028	0.567	3.448	2.186	0.419	2506.273
σ^2^_E_	25.317	0.239	0.522	1.185	2.228	2.139	0.223	0.019	3.484	0.002	0.010	0.002	0.006	0.013	0.002	17.058
σ^2^_P_	1232.918	2.487	14.595	19.044	109.096	50.876	5.406	0.819	95.732	0.422	6.038	0.569	3.454	2.199	0.422	2523.331
σ^2^_GCA_	1.021	0.002	0.517	0.005	0.219	0.006	0.148	16.411	1.175	0.088	−0.001	0.002	−0.004	−0.006	0.002	−0.004
σ^2^_SCA_	5.608	0.503	2.873	0.045	6.595	0.103	0.777	173.882	190.273	0.728	0.042	0.044	0.336	0.521	0.044	0.324
GA	0.182	0.004	0.180	0.111	0.033	0.058	0.190	0.094	0.006	0.121	−0.005	0.045	−0.012	−0.012	0.045	−0.012
RD	0.626	0.154	0.429	0.460	0.401	0.328	0.982	0.344	0.544	0.696	0.837	1.000	0.762	0.851	0.696	0.801
G _Adv._	1306.816	3.429	17.335	21.529	134.207	64.612	4.602	1.046	105.156	0.435	5.774	0.499	3.443	2.079	0.435	2449.537
H^2^	0.979	0.904	0.964	0.938	0.980	0.958	0.959	0.976	0.964	0.995	0.998	0.996	0.998	0.994	0.995	0.993
h^2^	0.602	0.783	0.675	0.642	0.699	0.722	0.484	0.726	0.624	0.587	0.543	0.498	0.566	0.537	0.587	0.552

*GP, germination percentage; DG, days to germination; DS, days to squaring; DF, days to flowering; DB, days to boll opening; PH, plant height (cm); NB, number of bolls per plant; BW, boll weight (g); YP, yield of seed cotton/ yield per plant (g); PC, proline content; ChA, chlorophyll a; ChB, chlorophyll b; ChT, chlorophyll ab or total chlorophyll; ChAB, chlorophyll a/b;CTe, canopy temperature; CMS, cell membrane stability of 31 cotton genotypes including 10 parents and 21 crosses. σ^2^_L_, line variance; σ^2^_T_, tester variance; σ^2^_LxT_, line x tester variance; σ^2^_Gen_., genotype variance; σ^2^_A_, additive variance; σ^2^_D_, dominance variance; $\sigma_{\text{G}}^{2}$, genetic variance; σ^2^_E_, environmental variance; σ^2^_P_, phenotypic variance; σ^2^_GCA_, GCA variance; σ^2^_SCA_, SCA variance; GA, gene action; RD, rate of dominance; G Adv., genetic advance; H^2^, broad heritability; h^2^, narrow heritability*.

The proportional contribution of genotypes including lines, testers, and cross combinations for studied traits toward total variance (Figure 4) was evident that lines had a significant contribution in total variance for DBO of 19 and 10% under NS and DS, respectively, which indicates that lines contributed more positive alleles for phenological traits, so these traits might be under the maternal influence. Lines had significantly higher variance contribution for PC of 13% under DS conditions. At the same time, Testers were more important for BW (14%) and CMS (13%), indicating that testers are more important for these traits among the studied traits. Contribution of the cross combination was significantly higher for ChAB (10%), ChA (9%), and ChB (10%) under DS conditions.

### Mean Performance of Parents and Cross Combinations

**Figure 5:**
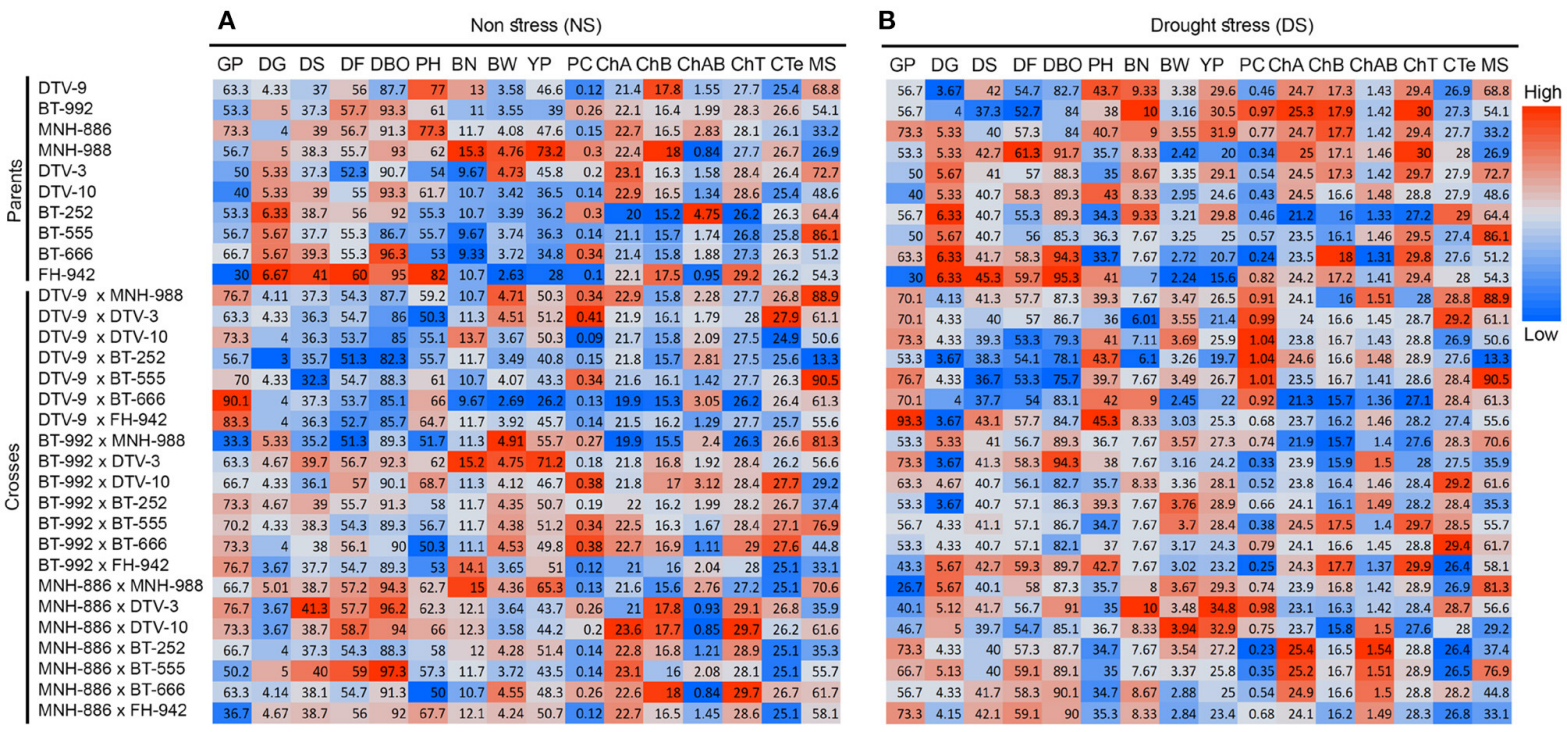
Mean performance of 10 parents and 21 cross combinations under **(A)** NS and **(B)** DS environmental conditions. The magnitude of trait values increases from low (blue) to high (red).

**Figure 6:**
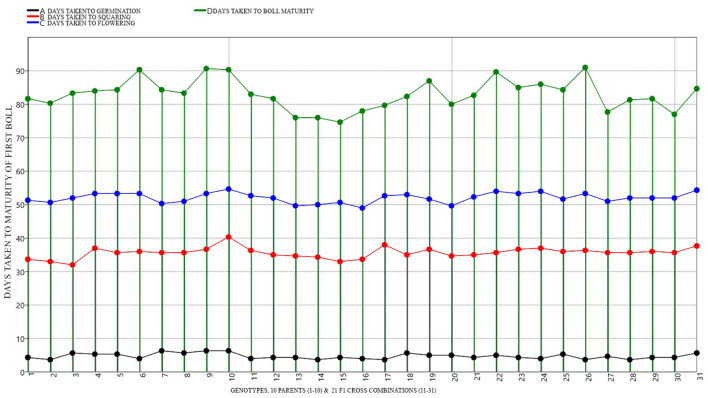
Graphical representation and mean performance of maturity traits among 10 parents (1-10) and 21 cross combinations (11-31) under DS environmental conditions.

Expectedly, significant variability in the performance of parents and crosses was noticed. Differential performance of parents and crosses was compared under both NS and DS conditions (Figures 5, 6 and Supplementary Tables S8–S11). DTV-9, BT-992, MNH-886 among the parents had low DBO days of 82.67, 84, and 84 respectively, under DS. Meanwhile, under NS there was no significant difference in maturity days among all parents. Among the crosses, DTV-9 × DTV-10, DTV-9 × BT-555, and DTV-9 × BT-666 had the least DBO of 79.33, 78.12, and 75.67, respectively, under DS, which was significantly higher under NS conditions. Six hybrid combinations exhibited early maturity within less than 80 days while nine stranded combinations exhibited late mature genotypes with 91 days after sowing (Figure 6).

Yield and yield contributing trait values for YP, NB, and BW were also higher in lines including DTV-9, BT-992, MNH-886 among the parents, under DS conditions, while among the crosses, MNH-886 x DTV-10 and MNH-886 × BT-252 had significantly high YP, BW, and BN under DS conditions. In the physiological traits, including chA, CHB, and ChT were higher in drought-tolerant lines DTV-9, BT-992, MNH-886 under DS conditions.

### Field Performance of Identified Parent Genotypes Under Rainfed Conditions

**Figure 7:**
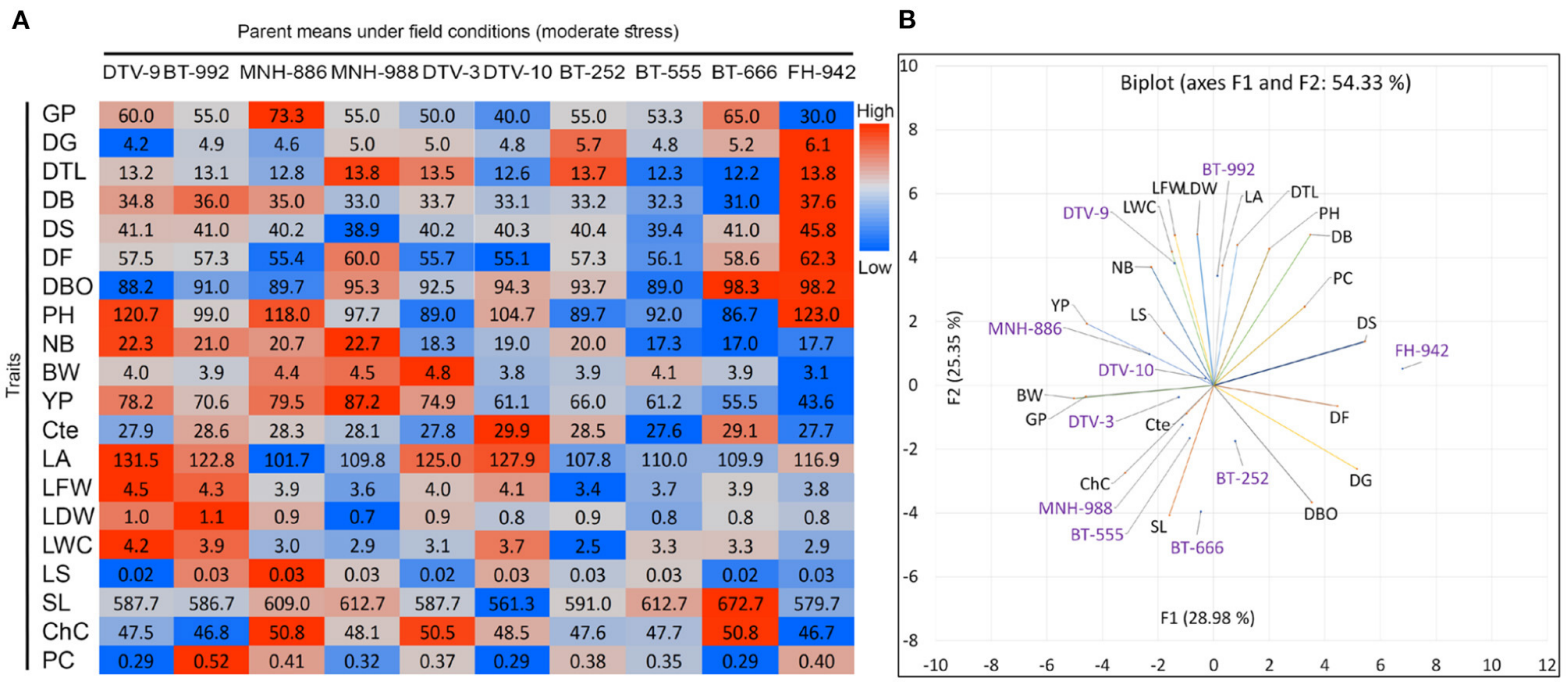
Field performance of 10 parent genotypes under rainfed conditions. **(B)** Biplot and heatmap of mean values of morpho-physiological and **(A)** phenological traits at maturity stage under field conditions.

A significant variation was observed in the mean performance of 10 parents under field conditions (Figure 7). The distribution of genotypes and traits is indicating a high variability of 54.33% for components 1 (28.98%) and 2 (25.35%) in PCA biplot (Figure 7). DTV_9, BT-992, and MNH-886 were performed well under field conditions for YP and NB. In terms of phenological traits, MNH-889 had minimum DS and DTV-10 had minimum DF but also had high values for DBO as compared with DTV_9, BT-992, and MNH-886. DTV_9, BT-992, and MNH-886 secure significant high values for physiological traits, including LWC, LS, and PC. DTV_9 had maximum LA, LFW, and LWC. Lastly, FH_942 had maximum values for phenological traits with the lowest GP, PY, and other yield contributing traits. Despite this, it also had the lowest ChC.

## Discussions

### The Basis of Selection for Parent Genotypes

A noticeable variation was found in the panel of 16 cotton genotypes for all morpho-physiological and biochemical parameters with some exceptions under DS condition, which suggested the genetic potential of improvement for drought tolerance in studied material (Supplementary Table S2 and Figures 2, 3).

Healthy plant seedlings have a significant role in crop yield performance, but drought stress unswervingly reduce the growth of plants by affecting their normal growth at early seedling stages (Dugasa et al., 2019). A significant reduction in plant total biomass (PTDW), including other morphological traits was observed under the DS condition, which indicates the reduction in nutrient uptake from the soil due to the low water potential of soil under osmotic stress. Meanwhile, less water and nutrient uptake affect plant membrane stability and permeability (van Bavel, 1996). Such nutrition imbalances forced the plant to stunt its normal growth by enhancing the nutrient uses compared to nutrient uptake and energy resources, which causes a reduction in the plant total biomass (Hu et al., 2006). In the results, a severe decline in plant biomass, cell membrane stability, including various morpho-physiological traits, was observed under DS conditions, which was also consistent with the results of Hassan et al. (2015).

Chlorophyll content accumulation was observed in MNH-886, BT-992, and DTV-9, increased RWC in BT-992 and less excise leaf water content (ELWC) in MNH-886 and DTV-9 was also noticed under DS condition, which indicates that drought-tolerant genotypes adapt stomatal closure strategy and accumulates more relative water contents coupled with less excise water in their leaf tissue to maintain the photosynthetic activities as compared to susceptible genotypes (Chaves et al., 2009). Drought stress circumstances create a huge and rapid decline in chlorophyll A, B, and total contents which ultimately reduce photosynthate production and is a reason behind the stunted growth of plants under drought stress (Manivannan et al., 2007). The low value of chlorophyll A and B ratio is the best criteria to select tolerant accession. The result for chlorophyll A/B ratio is similar to the results of Ali et al. (2009), which depicted that the tolerant genotypes having less ELC with high photosynthesis and relative water content than susceptible accessions. The results for RWC, ELW, and chlorophyll contents are also in line with the results of Nyachiro et al. (2001).

Reduction in photosynthesis also enhanced the production of reactive oxygen species (ROS) including H_2_O_2_ and ${\text{O}}_{2}^{-}$ (Zafar et al., 2018). These elements badly damage the cell membrane and proteins under oxidative stress. Under DS conditions, plants use antioxidant machinery to reduce oxidative stress by enzymatic and non-enzymatic activities (Alscher et al., 2002). To compensate for the effect of H_2_O_2_ and ${\text{O}}_{2}^{-}$, plants trigger SOD as a defense compound in mitochondria, chloroplast, nuclei, and apoplast (Gill and Tuteja, 2010). In response to drought stress, SOD increases which directly increases the level of CAT and then CAT, SOD, and APX coupled with glutathione reductase (GR) enzyme convert H_2_O_2_ and ${\text{O}}_{2}^{-}$ into water and O^2^ (Ghabooli et al., 2013).

Similarly, in the results, induction and the relative increase in anti-oxidants including SOD, NOX, POX, GR, and CAT was noticed under DS condition which was in line with the results of Pan et al. (2006). The results depicted that the tolerant genotypes including MNH-886, BT-992, and DTV-9 have better antioxidant based defensive system than susceptible genotypes. Meanwhile, in tolerant genotypes, a significant increase in proline contents and carotenoids was observed which indicates that these non-enzymatic elements act as safeguard to chloroplast through dissipating increased excitation energy under stress (Parida et al., 2004; Moore et al., 2016). Both enzymatic and non-enzymatic processes are facilitated by osmotic balance during drought stress in tolerant genotypes. Osmotic adjustment is maintained by the accumulation of cell solutes such as proline contents. At the same time, proline accumulation in plant tissues reduces cell death and cell membrane injury (Mahmood et al., 2020a).

Results of the screening experiment are significantly reliable and encouraging to investigate further the inheritance pattern of drought tolerance in identified drought-tolerant (DTV-9, BT-992, and MNH-886) and susceptible (FH-942, BT-666, MNH-988, DTV-10, BT-252, and BT-142) genotypes.

### The Inheritance Pattern of Drought Tolerance in Cotton

Drought tolerance is complex traits, so the environment and genotypic effect on phenotype cannot always be independent. Thus, genotype × environment interaction may have a masking effect on phenotype inheritance. The heterozygosity and homozygosity of different genotypes respond contrarily under different environments (Cole et al., 2009).

#### Combining Ability (GCA and SCA) Effects of Parents and Cross Combinations

Parental genotypes, including lines and testers, both displayed considerable variation in GCA effects across the environmental conditions in desired directions with limited exceptions. Under DS condition, more significant GCA effects were observed as compared with NS conditions, which indicates more variability of studied traits is present among all parents. It can be utilized in the hybridization for drought-tolerant variety development similar to the results that were previously reported by Abid et al. (2016). Traits with more differential GCA effects among the lines and testers can be considered candidate traits for breeding drought-tolerant cotton. The traits, including DBO among phenological traits, BW among yield traits and PC, ChA, ChAB ratio, and ChT among physiological traits, had more significant GCA effects under DS condition as compared with NS condition. It indicates the importance of these traits as candidate traits for the development of drought-tolerant cotton. Results also revealed that DTV-9 and FH-942 are the best general combiner line and tester, respectively, with significant GCA effects for the maximum number of traits. Parents with desirable and higher GCA effects for favorable traits might have contributed to more additive variance under DS conditions. It had been previously reported that genotypes with improved performance under DS condition could result from the contribution of more favorable alleles by parents (Ertiro et al., 2017). Meanwhile, under both NS and DS conditions, the contribution of dominance variance for most of the traits was significantly higher which suggested to exploit both components for breeding programs by evaluating the parents for GCA followed by the testing of their cross combinations under targeted environmental conditions due to the difference in effects of the genetic basis of GCA and SCA (Makumbi et al., 2011; Qi et al., 2013).

Crosses displayed remarkable variation in the SCA effects across the environments in both positive and negative directions. Several combinations had more significant SCA effects for PH, BN, BW, YP, CTe, and CMS under the NS condition as compared with DS. Meanwhile, under DS conditions, several combinations had significant SCA effects for phenological and physiological traits including GP, DS, DBO, PC, ChA ChB, ChAB, ChT, and CMS, which suggested the possibility of identifying specific combinations that can perform well under various conditions, as it had been explained by Patil et al. (2018) and Ertiro et al. (2017).

Under DS condition, among all the cross combinations, DTV-9 × BT-252 and DTV-9 × DTV-10 had maximum negative SCA effects for DBO. Simultaneously, DBO also had higher heritability (h^2^) and dominant gene action which indicates the importance of these combinations and traits simultaneously for the early mature and short duration variety development. Among the phenological traits, early boll ripening is a strong indicator of early maturity and the short duration of the cotton crop (Bednarz and Nichols, 2005; Liu et al., 2015). BT-992 × FH-942 had maximum positive SCA effects for ChT and ChB. Simultaneously, it also had a higher positive SCA effect for CMS. It was also observed that BT-992 × FH-942 had significant SCA effects for a maximum of 8 traits which also indorse the selection criteria of hybrids for drought-tolerant cotton breeding. BT-992 × BT-666 and MNH-886 × DTV-10 had maximum significant SCA effects for BW and YP respectively, which may be the heritance effect of parent lines BT-992 and MNH-886 because these two lines have maximum plant yield under drought stress environment.

Additionally, these lines are also the high yielding commercial varieties of Pakistan. The SCA affects between lines and testers can be used as an indicator of PY under the same environmental conditions (Makumbi et al., 2011; Memon, 2017). On the other hand, SCA among the environmental conditions was not related to the positive relationship of mean GY among the different environmental conditions and the preponderance of additive genetic variance may have been in control of grain yield as it was exploited by Ertiro et al. (2017).

#### Heritability of Drought Tolerance and Possible Gene Action for Studied Traits

Knowledge about the inheritance pattern of targeted traits, including heritability and gene action is important for setting a breeding strategy to improve drought tolerance in the targeted materials. For a specific trait, the amount of dominance and additive variance components is important and suggests the scope of selection to develop drought-tolerant hybrids with enhanced stress tolerance. The higher proportional contribution of additive variances for YP and yield contributing traits, including BW and BN, suggests the additive gene action. Improvement and selection based on such traits can be fruitful through a recurrent selection scheme rather than natural selection (Ertiro et al., 2017).

The negative variance of general combining ability (σ^2^GCA) for some characters indicates an additive type of gene action in those traits. Variances of specific combining ability (σ^2^SCA) were higher in magnitude than the corresponding variances of general combining ability (σ^2^GCA) for all the traits. This indicated the preponderance of non-additive gene action in their inheritance which might be the result of dominance, epistasis, and interaction effects (Farooq et al., 2019). Among the phenological trait, DS and DBO have a significantly higher heritability with a higher genetic advance which indicates the potential of improvement of early maturity in cotton, significance, and potential of early maturity that has also been reported (Bednarz and Nichols, 2005; Li et al., 2017). Getting early maturity and a shortened growth period for avoiding the stress period have also been explored in other crops like wheat (Aziz et al., 2018). Interestingly, fewer differences among H^2^ and h^2^ of yield traits including YP and BW have been noticed as compared with physiological traits under DS conditions, which indicates the less environmental influence on such traits, which is also well-reported previously (Ulloa, 2006).

Lines also had higher variance contribution for DF under NS and DS conditions, which indicates that lines contributed more positive alleles for phenological traits so that these traits might be under the maternal influence. Lines had significantly higher variance contribution for PC under DS conditions. While, in testers, more contributions for BW and CMS were observed. The above results indicate that these parents are more important for these traits and can be used as the source of the genetic basis for drought tolerance (Farooq et al., 2019).

#### Mean Performance and Genetic Advance Among Parents and Cross Combinations

Selection of suitable parents for hybrid breeding based on combining ability effects, alone, has limited value. Therefore, the selection of parents based on GCA and SCA effects coupled with their performance would be of great value. In the differential performance of parents and crosses, significant variability was noticed under both NS and DS conditions (Figures 3, 4).

Short growth period and early maturity characters including days taken to first square (DS), days taken to first flower (DF), and days have taken to days to first boll open (DBO) are very important to develop drought-tolerant cotton varieties (Li et al., 2017). Recently, it was also explained that early flowering improves drought tolerance in cotton (Guo et al., 2017). In the results, drought-tolerant genotypes showed early maturity and boll ripening, DTV-9 showed boll maturity in a minimum of 80 days after sowing, while susceptible genotypes showed delayed maturity. Interestingly, most of the hybrids displayed a maturity even earlier than the parents, indicating the high genetic advance and higher heritability of the early maturity traits of the parents. As expected, identified drought-tolerant genotypes, including DTV-9, BT-992, MNH-886, showed early maturity under DS as compared to NS condition, which indicates that early maturing genotypes can cop drought stress.

On the other hand, it also reflects that drought stress triggers early maturity in drought-tolerant genotypes (Kazan and Lyons, 2016). In the context of inheritance of early maturity traits, a cross result was found in which DTV-9 × DTV-10, DTV-9 × BT-555, and DTV-9 × BT-666 had taken a few days to reach maturity with high genetic advance and heritability (Figure 4). Under DS conditions, the inheritance of physiological traits was comparatively low, and the amount of heritability and the genetic advance were also lower than the other traits. Recently, Abid et al. (2016) suggested that selecting such traits will not be encouraging at early stages for breeding programs.

### Field Performance of Identified Parent Genotypes Under Rainfed Conditions

Field performance of 10 selected genotypes was tested under rainfed conditions considering mild drought stress. A hypothesis that short-duration and drought-tolerant varieties can perform better under rainfed conditions without supplement irrigations was proposed. It was well-reported previously that early flowering and maturity improve drought tolerance in cotton (Luo et al., 2016; Guo et al., 2017). As expected, identified drought-tolerant genotypes including DTV_9, BT-992, and MNH-886 performed well under field conditions for yield, yield-related traits, and other physiological traits including LWC, LS, and PC, which are the strong indicators of drought tolerance (Hussain et al., 2019; Mahmood et al., 2020a). FH-942 stood as a late maturing variety with the lowest germination and plant yield that may have low chlorophyll contents because of low plant yield under field conditions due to chlorophyll content being directly correlated with plant yield in cotton (Karademir et al., 2009). These traits influenced environment and genotype × environment inter-action (Rosielle and Hamblin, 1981; Luo et al., 2016).

### Future Perspectives and Recommendations

The narrow genetic basis and low quality of seed are among the significant reasons for reducing cotton yield. Current cultivars cannot tolerate uncertain weather conditions and insect-pest infestation. Limited cotton-growing areas and the narrow genetic basis of the current cotton cultivars are the major reasons for a huge cotton yield reduction (Supplementary Table S12 and Supplementary Figure S1). New potential areas that have favorable climatic conditions can be explored for cotton growth and production. Screening and breeding new varieties that have more tolerance against abiotic and biotic stresses are the possible solutions to overcome the yield losses. In this study, the agro-climatic zones and cotton-growing areas of Pakistan were explored. New areas for cotton cultivation, which have a huge potential for cotton cultivation and suitable agroclimatic conditions for cotton cultivation (Figure 1), are recommended. Here, new potential areas of the upper Punjab and south Khaiber Pakhton Khan (KPK) is proposed and suggested for cotton cultivation after performing a set of experiments in one of these areas (potohar region) as they were observed to have almost the same agro-climatic conditions. Districts of upper Punjab and lower KPK with summer mean temperatures between 30 and 35 are the most suitable potential areas for commercial cotton cultivation (Figure 1).

## Conclusion

Global climate changes and uncertainty of weather is a serious threat to cotton production and yield. The study was designed to identify drought-tolerant genotypes of cotton and explore the potential and inheritance pattern of drought tolerance in cotton. Considerable possibilities of improvement and high heritability of studied traits under drought stress environments were found. Identified cotton genotypes including DTV-9, BT-992, and MNH-886 can be directly used for cultivation under moderate drought stress conditions. These lines have a huge potential for early maturity and drought tolerance as the early maturity traits had a significant heritability, which can be utilized in future breeding programs to develop a useful plant ideotype to encounter the climate shift globally. Our findings and approaches to use the variability of drought tolerance to cope with the situation can be the baseline to design future studies to develop high yielding and drought tolerant cotton verities. Meanwhile, there is a huge need for new drought tolerant verities with unique features including dwarf plant type, early maturity, high boll weight with good fiber qualities and a strong physiological mechanism with high water use efficiency. A comprehensive genome-wide association study is needed to exploit the genetic basis of drought tolerance in cotton. In a broader vision, other solutions beyond the traditional and practicing approaches should be explored which will ultimately, give us a broader picture to understand the drought tolerance behavior of cotton.

## Data Availability Statement

The original contributions presented in the study are included in the article/Supplementary Material, further inquiries can be directed to the corresponding author/s.

## Author Contributions

TM and MS conceived and designed the article. FM-P, TM, XW, SA, MA, RR, MY, SK, TJ, JT-C, XD and MSI contributed to the manuscript revision. FM-P, TM, XW, SA, MA, RR, MY, SK, TJ, JT-C, XD and MSI wrote the manuscript and supervision XD and MS. All authors contributed to the article and approved the submitted version.

## Funding

The publication of the present work is supported by the Natural Science Basic Research Program of Shaanxi Province (grant no. 2018JQ5218) and the National Natural Science Foundation of China (51809224), Top Young Talents of Shaanxi Special Support Program and the Chilean National Fund for Scientific and Technological Development (FONDECYT) grant number 1201973.

## Conflict of Interest

The authors declare that the research was conducted in the absence of any commercial or financial relationships that could be construed as a potential conflict of interest.

## Publisher's Note

All claims expressed in this article are solely those of the authors and do not necessarily represent those of their affiliated organizations, or those of the publisher, the editors and the reviewers. Any product that may be evaluated in this article, or claim that may be made by its manufacturer, is not guaranteed or endorsed by the publisher.
