# Supplementary material for: Genetic Potential and Inheritance Pattern of Phenological Growth and Drought Tolerance in Cotton (Gossypium Hirsutum L.)
Source: Front Plant Sci. 2021 Sep 24;12:705392. doi: 10.3389/fpls.2021.705392 (PMC8497812; doi:10.3389/fpls.2021.705392)
Supplement: Supplementary file 1 [file Data_Sheet_1.docx]

**Supplementary Table S1**. List of genotypes and cross combinations used in the study

| **Sr#** |  | **Lines** | **Tester** | **Lines** | **Tester** | **Genotype** |
| --- | --- | --- | --- | --- | --- | --- |
| 1 |  | 1 |  | DTV-9 |  | P-1 |
| 2 |  | 2 |  | BT-992 |  | P-2 |
| 3 |  | 3 |  | MNH-886 |  | P-3 |
| 4 |  |  | 1 |  | MNH-988 | P-4 |
| 5 |  |  | 2 |  | DTV-3 | P-5 |
| 6 |  |  | 3 |  | DTV-10 | P-6 |
| 7 |  |  | 4 |  | BT-252 | P-7 |
| 8 |  |  | 5 |  | BT-555 | P-8 |
| 9 |  |  | 6 |  | BT-666 | P-9 |
| 10 |  |  | 7 |  | FH-942 | P-10 |
| 11 |  | 1 | 1 | DTV-9 | MNH-988 | H-1 |
| 12 |  | 1 | 2 | DTV-9 | DTV-3 | H-2 |
| 13 |  | 1 | 3 | DTV-9 | DTV-10 | H-3 |
| 14 |  | 1 | 4 | DTV-9 | BT-252 | H-4 |
| 15 |  | 1 | 5 | DTV-9 | BT-555 | H-5 |
| 16 |  | 1 | 6 | DTV-9 | BT-666 | H-6 |
| 17 |  | 1 | 7 | DTV-9 | FH-942 | H-7 |
| 18 |  | 2 | 1 | BT-992 | MNH-988 | H-8 |
| 19 |  | 2 | 2 | BT-992 | DTV-3 | H-9 |
| 20 |  | 2 | 3 | BT-992 | DTV-10 | H-10 |
| 21 |  | 2 | 4 | BT-992 | BT-252 | H-11 |
| 22 |  | 2 | 5 | BT-992 | BT-555 | H-12 |
| 23 |  | 2 | 6 | BT-992 | BT-666 | H-13 |
| 24 |  | 2 | 7 | BT-992 | FH-942 | H-14 |
| 25 |  | 3 | 1 | MNH-886 | MNH-988 | H-15 |
| 26 |  | 3 | 2 | MNH-886 | DTV-3 | H-16 |
| 27 |  | 3 | 3 | MNH-886 | DTV-10 | H-17 |
| 28 |  | 3 | 4 | MNH-886 | BT-252 | H-18 |
| 29 |  | 3 | 5 | MNH-886 | BT-555 | H-19 |
| 30 |  | 3 | 6 | MNH-886 | BT-666 | H-20 |
| 31 |  | 3 | 7 | MNH-886 | FH-942 | H-21 |

**Supplementary Table S2.** Drought stress response indices of 25 physio-morphological and biochemical traits at seedling stage.

| No | Gen | EWC | RWC | Cha | Chb | Chab | Cht | BeC | SL | RL | R/S | RDW | RFW | PTDW | SFW | SDW | PTFW | PC | H2O2 | SOD | NOX | CAT | POX | APX | GR | CDSRI |
| --- | --- | --- | --- | --- | --- | --- | --- | --- | --- | --- | --- | --- | --- | --- | --- | --- | --- | --- | --- | --- | --- | --- | --- | --- | --- | --- |
| 1 | IR-NIBGE 1524 | 0.38 | 0.41 | 0.92 | 0.56 | 1.40 | 1.59 | 0.16 | 0.88 | 0.96 | 1.05 | 0.32 | 0.24 | 0.22 | 0.90 | 0.40 | 0.49 | 3.74 | 2.82 | 2.32 | 1.82 | 1.60 | 1.88 | 2.35 | 1.02 | 28.44 |
| 2 | BT-992 | 0.40 | 0.51 | 0.94 | 0.90 | 2.52 | 1.06 | 0.13 | 0.96 | 1.09 | 1.32 | 0.41 | 0.44 | 0.38 | 1.05 | 0.51 | 0.62 | 3.30 | 3.04 | 1.53 | 3.15 | 1.65 | 3.29 | 3.57 | 1.57 | 34.33 |
| 3 | IR-NIBGE-3701 | 0.41 | 0.59 | 1.05 | 0.55 | 3.63 | 1.07 | 0.10 | 1.01 | 1.14 | 1.15 | 0.54 | 0.59 | 0.49 | 1.10 | 0.59 | 0.73 | 2.33 | 3.10 | 1.60 | 2.42 | 1.63 | 2.36 | 2.82 | 1.21 | 32.19 |
| 4 | IR-NIBGE-901 | 0.28 | 0.27 | 0.77 | 0.78 | 1.78 | 1.12 | 0.12 | 0.88 | 0.94 | 1.13 | 0.12 | 0.16 | 0.22 | 0.90 | 0.47 | 0.46 | 2.42 | 3.45 | 1.99 | 2.44 | 1.64 | 2.51 | 3.28 | 1.41 | 29.56 |
| 5 | NIBGE-115 | 0.29 | 0.24 | 0.98 | 0.55 | 2.75 | 0.86 | 0.12 | 0.92 | 0.98 | 1.02 | 0.11 | 0.28 | 0.25 | 0.90 | 0.42 | 0.48 | 2.69 | 3.38 | 1.76 | 2.61 | 1.64 | 2.15 | 3.46 | 1.40 | 30.24 |
| 6 | IR-NIBGE-3 | 0.41 | 0.32 | 0.96 | 0.65 | 2.09 | 1.49 | 0.19 | 1.02 | 1.13 | 1.29 | 0.17 | 0.20 | 0.18 | 1.06 | 0.50 | 0.53 | 2.78 | 3.17 | 1.22 | 1.94 | 1.60 | 2.19 | 5.17 | 2.19 | 32.44 |
| 7 | BT-252 | 0.32 | 0.27 | 1.03 | 0.73 | 3.30 | 0.89 | 0.14 | 0.96 | 0.96 | 1.01 | 0.40 | 0.28 | 0.25 | 0.89 | 0.42 | 0.47 | 1.50 | 2.86 | 1.59 | 1.23 | 1.58 | 1.50 | 2.12 | 0.86 | 25.55 |
| 8 | MNH-988 | 0.24 | 0.23 | 0.79 | 0.58 | 3.07 | 1.83 | 0.18 | 0.86 | 0.93 | 0.97 | 0.21 | 0.18 | 0.18 | 0.89 | 0.42 | 0.44 | 1.05 | 2.91 | 1.18 | 0.98 | 1.55 | 1.19 | 4.31 | 1.69 | 26.85 |
| 9 | FH-942 | 0.25 | 0.23 | 0.77 | 0.64 | 2.82 | 1.73 | 0.20 | 0.87 | 0.89 | 0.91 | 0.10 | 0.11 | 0.12 | 0.85 | 0.35 | 0.38 | 0.93 | 2.71 | 1.35 | 1.82 | 1.62 | 2.26 | 5.24 | 1.98 | 29.12 |
| 10 | BT555 | 0.28 | 0.26 | 0.78 | 0.65 | 2.79 | 1.22 | 0.16 | 0.79 | 0.91 | 1.10 | 0.26 | 0.19 | 0.18 | 0.87 | 0.38 | 0.43 | 2.50 | 3.35 | 1.55 | 2.20 | 1.62 | 1.97 | 3.54 | 1.48 | 29.44 |
| 11 | BT-666 | 0.45 | 0.37 | 0.89 | 0.89 | 2.70 | 1.54 | 0.24 | 0.84 | 0.90 | 0.97 | 0.46 | 0.40 | 0.31 | 0.91 | 0.45 | 0.54 | 1.68 | 3.04 | 1.35 | 1.67 | 1.60 | 1.81 | 2.60 | 1.07 | 27.68 |
| 12 | DTV-10 | 0.46 | 0.46 | 1.05 | 0.76 | 1.06 | 1.52 | 0.16 | 0.95 | 1.06 | 1.32 | 0.38 | 0.54 | 0.44 | 1.07 | 0.55 | 0.67 | 1.49 | 2.74 | 1.68 | 1.31 | 1.54 | 1.45 | 2.46 | 1.07 | 26.17 |
| 13 | NN-3 | 0.36 | 0.43 | 0.94 | 0.80 | 2.61 | 0.93 | 0.16 | 0.88 | 0.94 | 1.04 | 0.47 | 0.38 | 0.35 | 0.92 | 0.43 | 0.55 | 7.59 | 2.97 | 1.39 | 1.79 | 1.61 | 2.01 | 2.35 | 0.98 | 32.90 |
| 14 | MNH-886 | 0.26 | 0.25 | 0.86 | 0.65 | 2.26 | 1.23 | 0.18 | 0.84 | 0.95 | 1.01 | 0.14 | 0.20 | 0.27 | 0.91 | 0.44 | 0.50 | 1.50 | 2.70 | 1.35 | 4.37 | 1.68 | 3.11 | 5.04 | 2.13 | 32.85 |
| 15 | BT-142 | 0.31 | 0.28 | 1.16 | 0.52 | 1.75 | 0.87 | 0.14 | 0.96 | 1.12 | 1.26 | 0.20 | 0.19 | 0.26 | 1.02 | 0.42 | 0.52 | 1.67 | 2.61 | 1.51 | 1.43 | 1.54 | 1.49 | 2.67 | 1.17 | 25.07 |
| 16 | DTV-9 | 0.27 | 0.25 | 0.80 | 0.88 | 1.90 | 1.71 | 0.20 | 0.86 | 0.93 | 1.14 | 0.06 | 0.09 | 0.23 | 0.88 | 0.44 | 0.46 | 4.57 | 3.16 | 1.02 | 3.87 | 1.67 | 3.85 | 4.84 | 2.07 | 36.16 |

**Supplementary Table S3.** GCA of lines and testers (Parents) under NS conditions

| LINES | GP | DG | DS | DF | DBO | PH | BN | BW | YP | PC | ChA | ChB | ChAB | ChT | CTe | CMS |
| --- | --- | --- | --- | --- | --- | --- | --- | --- | --- | --- | --- | --- | --- | --- | --- | --- |
| DTV-9 | 6.51 ** | -0.27ns | -1.57** | -1.57 ** | -4.03 ** | -0.05 ns | -0.62 ** | -0.24 ** | -5.15 ** | 0.00 ns | -0.20 ** | -0.44 ** | 0.24 ns | -0.64 ** | 0.00 ns | 4.98 ** |
| BT-992 | -1.59 ns | 0.21ns | 0.14 ns | -0.05 ns | 0.44 ns | -1.67 ** | 0.33 ns | 0.28 ** | 4.65 ** | 0.04 * | -0.01 ns | 0.00 ns | 0.17 ns | -0.01 ns | 0.04 ** | -3.88 * |
| MNH-886 | -4.92 * | 0.06ns | 1.43* | 1.62 ** | 3.59 ** | 1.71 ** | 0.29 ns | -0.05 ns | 0.50 ns | -0.05 ** | 0.21 ** | 0.44 ** | -0.42 ns | 0.65 ** | -0.05 ** | -1.09 ns |
| TESTERS |  |  |  |  |  |  |  |  |  |  |  |  |  |  |  |  |
| MNH-988 | -7.94* | 0.56ns | -0.52ns | -0.92 ns | 0.70 ns | -1.08 ns | 0.38 ns | 0.56 ** | 8.00 ** | 0.02 ns | -0.37 ** | -0.66 ** | 0.62 ns | -1.03 ** | 0.02 ** | 25.04** |
| DTV-3 | 0.95 ns | -0.00ns | 1.59** | 1.19 * | 1.70 * | -0.63 ns | 0.83 ** | 0.20 * | 6.28 ** | 0.06 ** | 0.07 ns | 0.30 * | -0.32 ns | 0.36 ns | 0.06 ** | -4.00 ns |
| DTV-10 | 4.29ns | -0.22ns | -0.52ns | 1.30 ** | -0.08 ns | 4.37 ** | 0.49 ns | -0.31 ** | -2.05 ns | -0.00 ns | 0.05 ns | 0.36 * | 0.16 ns | 0.41 * | -0.00 ns | -8.05* |
| BT-252 | -1.27ns | -0.33ns | -0.19ns | -1.37 ** | -2.41 ** | -1.63 * | -0.17 ns | -0.06 ns | -1.49 ns | -0.06 ** | 0.16 ns | -0.07 ns | 0.14 ns | 0.09 ns | -0.06 ** | -26.56** |
| BT-555 | -3.49ns | 0.33ns | -0.63ns | 0.86 ns | 1.92 * | -0.52 ns | -0.62 * | -0.04 ns | -3.12 * | 0.05 ** | 0.12 ns | -0.15 ns | -0.14 ns | -0.04 ns | 0.05 ** | 19.17 * |
| BT-666 | 8.73** | -0.22ns | 0.25ns | -0.37 ns | -0.97 ns | -3.41 ** | -1.51 ** | -0.18 * | -7.68 ** | 0.03 ns | -0.09 ns | 0.31 * | -0.20 ns | 0.22 ns | 0.03 ** | 0.70 ns |
| FH-942 | -1.27ns | -0.11ns | 0.03ns | -0.70 ns | -0.86 ns | 2.92 ** | 0.60 * | -0.17 ns | 0.05 ns | -0.10 ** | 0.06 ns | -0.08 ns | -0.27 ns | -0.02 ns | -0.10 ** | -6.30 * |
| GCA Var. | -3.204 | 0.002 | 0.078 | 0.145 | 0.858 | -0.692 | 0.006 | 0.001 | 1.648 | 0 | 0.0008 | 0.012 | -0.001 | 0.022 | 0 | 16.411 |

**Supplementary Table S4.** GCA of lines and testers (Parents) under DS condition

| LINES | GP | DG | DS | DF | DBO | PH | BN | BW | YP | PC | ChA | ChB | ChAB | ChT | CTe | CMS |
| --- | --- | --- | --- | --- | --- | --- | --- | --- | --- | --- | --- | --- | --- | --- | --- | --- |
| DTV-9 | 11.11** | -0.40* | -0.97 ** | -1.44 ** | -3.89 ** | 3.00 ** | -0.46 * | -0.08 * | -2.27 ** | 0.25 ** | -0.11 ** | -0.09 ** | -0.06 ns | -0.20 ** | 0.25 ** | 4.98 ** |
| BT-992 | -4.60* | 0.08ns | 0.70 * | 0.60 ns | 1.30 * | -0.29 ns | -0.08 ns | 0.04 ns | 0.12 ns | -0.17 ** | 0.03 ns | 0.12 ** | 0.06 ns | 0.16 ** | -0.17 ** | -1.09 ns |
| MNH-886 | -6.51** | 0.32ns | 0.27 ns | 0.84 * | 2.59 ** | -2.71 ** | 0.54 ** | 0.08** | 2.16 ** | -0.08 ** | 0.07 ** | -0.03 ns | 0.00 ns | 0.04 ns | -0.08 ** | -3.88 * |
| TESTERS |  |  |  |  |  |  |  |  |  |  |  |  |  |  |  |  |
| MNH-988 | -11.27** | 0.56* | 0.33 ns | 0.71 ns | 2.02 * | -0.78 ns | -0.06 ns | 0.21 ** | 1.51 ns | 0.10 ** | -0.06 * | -0.28 ** | 0.48 ** | -0.34 ** | 0.10 ** | 25.04 ** |
| DTV-3 | -0.16ns | -0.11ns | 0.56 ns | 0.60 ns | 4.68 ** | -1.67 * | 0.05 ns | 0.05 ns | 0.61 ns | 0.10 ** | 0.05 ns | -0.19 ** | 0.19 * | -0.14 * | 0.08 * | -4.00 ns |
| DTV-10 | -0.16ns | 0.22ns | -0.56 ns | -2.06 ** | -3.65 ** | -0.22 ns | 0.05 ns | 0.31 ** | 2.72 * | 0.08 ** | -0.07 * | -0.17 ** | 0.13 ns | -0.24 ** | 0.08 * | -8.05 ** |
| BT-252 | -1.27ns | -0.56* | -0.78 ns | -0.62 ns | -1.98 * | 1.22 ns | -0.73 * | 0.17 * | -0.94 ns | -0.05 ns | 0.12 ** | 0.01 ns | -0.08 ns | 0.12 * | -0.05 ns | -26.5** |
| BT-555 | 5.40ns | 0.11ns | -1.22 ** | -0.29 ns | -2.21 * | -1.56 * | -0.17 ns | 0.17 * | 0.77 ns | -0.11 ** | 0.08 ** | 0.51 ** | -0.47 ** | 0.60 ** | -0.11 ** | 19.17 ** |
| BT-666 | -1.27ns | -0.22ns | -0.44 ns | -0.29 ns | -0.98 ns | -0.11 ns | 0.60 * | -0.52 ** | -2.45 * | 0.06 * | -0.16 ** | -0.15 ** | -0.02 ns | -0.30 ** | 0.06 ns | 0.70 ns |
| FH-942 | 8.73** | -0.00ns | 2.11 ** | 1.94 ** | 2.13 * | 3.11 ** | 0.27 ns | -0.39 ** | -2.23 * | -0.16 ** | 0.03 ns | 0.27 ** | -0.22 ** | 0.31 ** | -0.16 ** | -6.30 * |
| GCA Var. | 1.175 | 0.006 | 0.088 | 0.148 | 1.021 | 0.517 | 0.002 | 0.005 | 0.219 | 0.002 | -0.0002 | -0.004 | -0.004 | -0.006 | 0.002 | 16.411 |

**Supplementary Table S5.** SCA of 21 hybrids for all studied traits under NS conditions

| CROSS | GP | DG | DS | DF | DBO | PH 6,7 | BN 4,4 | BW 6,5 | YP 4,4 | PC | ChA | ChB | ChAB | ChT | CTe | CMS |
| --- | --- | --- | --- | --- | --- | --- | --- | --- | --- | --- | --- | --- | --- | --- | --- | --- |
| 1 x 1 | 11.27 * | -0.51 ns | 1.90 * | 1.68 * | 1.25 ns | 1.27 ns | -1.05 * | 0.29 * | -1.64 ns | 0.09 ns | 0.63 ** | 0.64 * | -0.44 ns | 1.26 ** | 0.09 ** | 3.63 ns |
| 1 x 2 | -10.95 * | 0.38 ns | -1.21 ns | -0.10 ns | -1.41 ns | -7.84 ** | -0.83 ns | 0.45 ** | 0.95 ns | 0.12 ** | 0.21 ns | -0.07 ns | -0.00 ns | 0.13 ns | 0.12 ** | 4.96 ns |
| 1 x 3 | -4.29 ns | 0.27 ns | 0.90 ns | -1.21 ns | -0.63 ns | -8.17 ** | 1.84 ** | 0.12 ns | 8.34 ** | -0.14 ** | 0.01 ns | -0.41 ns | -0.17 ns | -0.40 ns | -0.14 ** | -1.50 ns |
| 1 x 4 | -15.40 ** | -0.62 ns | -0.10 ns | -0.87 ns | -0.97 ns | -1.51 ns | 0.51 ns | -0.31 * | -1.73 ns | -0.02 ns | 0.06 ns | -0.12 ns | 0.57 ns | -0.06 ns | -0.02 ns | -20.36 ** |
| 1 x 5 | 0.16 ns | 0.05 ns | -2.98 ** | 0.24 ns | 0.70 ns | 2.71 * | -0.05 ns | 0.24 ns | 2.47 ns | 0.06 ns | -0.17 ns | 0.40 ns | -0.54 ns | 0.24 ns | 0.06 ** | 11.16 * |
| 1 x 6 | 7.94 ns | 0.27 ns | 1.13 ns | 0.46 ns | 0.25 ns | 10.60 ** | -0.16 ns | -1.00 ** | -10.10 ** | -0.13 ** | -0.59 ** | -0.84 ** | 1.14 ns | -1.43 ** | -0.13 ** | 0.40 ns |
| 1 x 7 | 11.27 * | 0.16 ns | 0.35 ns | -0.21 ns | 0.81 ns | 2.94 * | 0.27ns | 0.22 ns | 1.70 ns | 0.01 ns | -0.14 ns | 0.40 ns | -0.55 ns | 0.26 ns | 0.01 ns | 1.72 ns |
| 2 x 1 | -23.97 ** | 0.35 ns | -2.14 * | -2.84 ** | -1.56 ns | -4.44 ** | -1.33 ** | -0.03 ns | -6.08 * | -0.02 ns | -0.56 ** | -0.17 ns | -0.26 ns | -0.73 * | -0.02 ns | 4.97 ns |
| 2 x 2 | -2.86 ns | 0.24 ns | 0.41 ns | 0.38 ns | 0.44 ns | 5.44 ** | 1.89 ** | 0.17 ns | 11.20 ** | -0.15 ** | -0.07 ns | -0.03 ns | 0.20 ns | -0.10 ns | -0.15 ** | 9.23 * |
| 2 x 3 | -2.86 ns | 0.13 ns | -1.14 ns | 0.60 ns | -0.11 ns | 7.11 ** | -1.44 ** | 0.05 ns | -5.02 * | 0.12 ** | -0.09 ns | -0.06 ns | 0.93 ns | -0.15 ns | 0.12 ** | -14.05 ** |
| 2 x 4 | 9.37 ns | 0.57 ns | 1.52 ns | 1.94 * | 3.56 ** | 2.44 ns | -0.44 ns | 0.02 ns | -1.57 ns | -0.01 ns | 0.00 ns | 0.02 ns | -0.19 ns | 0.02 ns | -0.01 ns | 12.64 ** |
| 2 x 5 | 8.25 ns | -0.43 ns | 1.30 ns | -1.62 * | -2.78 * | -0.00 ns | 0.00 ns | 0.04 ns | 0.55 ns | 0.03 ns | 0.19 ns | 0.14 ns | -0.23 ns | 0.33 ns | 0.03 * | 6.42 ns |
| 2 x 6 | -0.63 ns | -0.21 ns | 0.08 ns | 1.27 ns | 0.78 ns | -3.44 * | 0.22 ns | 0.33 * | 3.70 ns | 0.08 * | 0.41 * | 0.32 ns | -0.73 ns | 0.72 * | 0.08 ** | -7.26 ns |
| 2 x 7 | 12.70 * | -0.65 ns | -0.03 ns | 0.27 ns | -0.33 ns | -7.11 ** | 1.11 * | -0.57 ** | -2.78 ns | -0.05 ns | 0.13 ns | -0.22 ns | 0.27 ns | -0.09 ns | -0.05 ** | -11.96 ** |
| 3 x 1 | 12.70 * | 0.16 ns | 0.24 ns | 1.16 ns | 0.30 ns | 3.17 * | 2.38 ** | -0.25 ns | 7.72 ** | -0.07 * | -0.06 ns | -0.47 ns | 0.70 ns | -0.53 ns | -0.07 ** | -8.60 ns |
| 3 x 2 | 13.81 * | -0.62 ns | 0.79 ns | -0.29 ns | 0.97 ns | 2.40 ns | -1.06 * | -0.61 ** | -12.15 ** | 0.02 ns | -0.13 ns | 0.11 ns | -0.20 ns | -0.03 ns | 0.02 ns | -14.19 ** |
| 3 x 3 | 7.14 ns | -0.40 ns | 0.24 ns | 0.60 ns | 0.75 ns | 1.06 ns | -0.40 ns | -0.16 ns | -3.32 ns | 0.02 ns | 0.08 ns | 0.46 ns | -0.75 ns | 0.55 ns | 0.02 ns | 15.55 ** |
| 3 x 4 | 6.03 ns | 0.05 ns | -1.43 ns | -1.06 ns | -2.59 ns | -0.94 ns | -0.06 ns | 0.29 * | 3.30 ns | 0.03 ns | -0.07 ns | 0.10 ns | -0.38 ns | 0.04 ns | 0.03 * | 7.72 ns |
| 3 x 5 | -8.41 ns | 0.38 ns | 1.68 ns | 1.38 ns | 2.08 ns | -2.71 * | 0.05 ns | -0.29 * | -3.02 ns | -0.09 ** | -0.02 ns | -0.54 * | 0.77 ns | -0.57 ns | -0.09 ** | -17.58 ** |
| 3 x 6 | -7.30 ns | -0.06 ns | -1.21 ns | -1.73 * | -1.03 ns | -7.16 ** | -0.06 ns | 0.67 ** | 6.40 * | 0.05 * | 0.19 ns | 0.52 * | -0.41 ns | 0.71 * | 0.05 ** | 6.86 ns |
| 3 x 7 | -23.97 ** | 0.49 ns | -0.32 ns | -0.06 ns | -0.48 ns | 4.17 ** | -0.84 ns | 0.36 ** | 1.08 ns | 0.04 ns | 0.02 ns | -0.18 ns | 0.28 ns | -0.16 ns | 0.04 ** | 10.24 ns |
| SCA Var. | 200.3086 | 0.0228 | 1.9019 | 1.8454 | 1.9685 | 41.7706 | 1.6041 | 0.2476 | 50.737 | 0.0104 | 0.0896 | 0.1812 | 0.133 | 0.4471 | 0.0104 | 173.8821 |

**Supplementary Table S6.** SCA of 21 hybrids for all studied traits under DS conditions

| CROSS | GP 3,4 | DG 1,0 | DS 1,1 | DF 3,3 | DBO 5,4 | PH 1,2 | BN 2,1 | BW 3,3 | YP 2, | PC 6,7 | ChA 6,4 | ChB 8,9 | ChAB 6,7 | ChT 8,8 | CTe 7,7 | CMS 4,5 |
| --- | --- | --- | --- | --- | --- | --- | --- | --- | --- | --- | --- | --- | --- | --- | --- | --- |
| 1 x 1 3,2 | 8.89 ns | -0.60 ns | 1.52 * | 1.67 ns | 3.22 * | -0.89 ns | 0.35 ns | -0.02 ns | 1.06 ns | -0.13 * | 0.11 * | -0.09 ns | 0.05 ns | 0.02 ns | -0.13 * | 3.63 ns |
| 1 x 2 4,3 | -2.22 ns | 0.40 ns | -0.03 ns | 1.11 ns | -0.11 ns | -3.33 ** | -1.43 ** | 0.23 * | -3.13 ns | -0.02 ns | 0.10 * | 0.45 ** | -0.43 ** | 0.55 ** | -0.02 ns | 4.96 ns |
| 1 x 3 3,1 | 1.11 ns | 0.06 ns | 0.41 ns | 0.11 * | 0.89 * | 0.22 ns | -0.43 ns | 0.10 ns | -0.77 ns | 0.02 ns | 0.21 ** | 0.49 ** | -0.42 ** | 0.70 ** | 0.02 ns | -1.50 ns |
| 1 x 4 5,2 | -17.78 ** | 0.17 ns | -0.37 ns | -0.67 ns | -2.11 * | 1.44 ns | -0.65 ns | -0.19 ns | -3.28 ns | 0.15 ** | 0.13 ** | 0.30 ** | -0.15 ns | 0.43 ** | 0.15 ** | -20.36 ** |
| 1 x 5 4,3 | -1.11 ns | 0.17 ns | -1.59 * | -1.67 * | -4.22 ** | 0.22 ns | 0.46 ns | 0.04 ns | 1.98 * | 0.18 ** | -0.08 ns | -0.20 * | 0.11 ns | -0.28 * | 0.18 ** | 11.16 * |
| 1 x 6 2,4 | -1.11 ns | 0.17 ns | -1.37 ns | -1.00 ns | 1.89 ns | 1.11 ns | 1.02 * | -0.31 ** | 0.54 ns | -0.08 ns | -0.47 ** | -0.53 ** | 0.49 ** | -1.00 ** | -0.08 ns | 0.40 ns |
| 1 x 7 2,4 | 12.22 * | -0.38 ns | 1.41 ns | 0.44 ns | 0.44 ns | 1.22 ns | 0.68 ns | 0.14 ns | 3.60 ns | -0.11 * | 0.00 ns | -0.43 ** | 0.34 * | -0.43 ** | -0.11 * | 1.72 ns |
| 2 x 1 3,3 | 7.94 ns | 0.25 ns | -0.48 ns | -1.38 ns | 0.03 ns | -0.27 ns | -0.03 ns | -0.04 ns | -0.51 ns | 0.11 * | -0.11 * | -0.61 ** | 0.91 ** | -0.72 ** | 0.11 * | -8.60 ns |
| 2 x 2 4,4 | 16.83 ** | -0.75 ns | -0.37 * | 0.40 ns | 2.37 * | 1.95 * | -0.14 ns | -0.28 * | -2.75 ns | -0.27 ** | -0.07 ns | -0.50 ** | 0.52 ** | -0.57 ** | -0.27 ** | -14.19 ** |
| 2 x 3 3,3 | 6.83 ns | -0.08 ns | 0.08 ns | 0.73 ns | -0.97 ns | -1.83 ns | 0.52 ns | -0.35 ** | -1.02 ns | -0.08 ns | 0.04 ns | -0.02 ns | -0.30 * | 0.02 ns | -0.08 ns | 15.55 ** |
| 2 x 4 2,3 | -2.06 ns | -0.30 ns | 0.30 ns | 0.29 ns | 1.03 ns | 0.40 ns | 0.63 ns | 0.20 ns | 3.50 ns | 0.19 ** | -0.13 ** | -0.42 ** | 0.21 ns | -0.56 ** | 0.19 ** | 7.72 ns |
| 2 x 5 1,2 | -5.40 ns | -0.30 ns | 1.08 ns | -0.05 * | 1.59 ns | -1.49 ns | 0.08 ns | 0.14 ns | 1.29 ns | -0.03 ns | 0.03 ns | 0.46 ** | -0.34 * | 0.49 ** | -0.03 ns | -17.58 ** |
| 2 x 6 6,2 | -2.06 ns | 0.03 ns | -0.03 ns | -0.05 ns | -4.30 ** | -0.60 ns | -0.70 ns | 0.29 * | 0.41 ns | 0.21 ** | 0.18 ** | 0.21 * | -0.34 * | 0.40 ** | 0.21 ** | 6.86 ns |
| 2 x 7 5,3 | -22.06 ** | 1.14 * | -0.59 ns | 0.06 ns | 0.25 * | 1.84 ns | -0.37 ns | 0.02 ns | -0.92 ns | -0.12 * | 0.07 ns | 0.88 ** | -0.66 ** | 0.94 ** | -0.12 * | 10.24 * |
| 3 x 1 2,3 | -16.83 ** | 0.35 ns | -1.05 ns | -0.29 ns | -3.25 * | 1.16 ns | -0.32 ns | 0.06 ns | -0.55 ns | 0.03 ns | 0.00 ns | 0.70 ** | -0.95 ** | 0.70 ** | 0.03 ns | 4.97 ns |
| 3 x 2 5,1 | -14.60 ** | 0.35 ns | 0.40 ns | -1.51 * | -2.25 ns | 1.38 ns | 1.57 ** | 0.05 ns | 5.87 ** | 0.29 ** | -0.03 ns | 0.04 ns | -0.09 ns | 0.01 ns | 0.29 ** | 9.23 * |
| 3 x 3 2,4 | -7.94 ns | 0.02 ns | -0.49 ns | -0.84 ns | 0.08 * | 1.60 ns | -0.10 ns | 0.24 * | 1.79 ns | 0.06 ns | -0.25 ** | -0.47 ** | 0.71 ** | -0.72 ** | 0.06 ns | -14.05 ** |
| 3 x 4 2,2 | 19.84 ** | 0.13 ns | 0.06 ns | 0.38 * | 1.08 * | -1.84 ns | 0.02 ns | -0.02 ns | -0.21 ns | -0.33 ** | 0.00 ns | 0.13 ns | -0.06 ns | 0.13 ns | -0.33 ** | 12.64 ** |
| 3 x 5 0,3 | 6.51 ns | 0.13 ns | 0.51 * | 1.71 * | 2.63 ns | 1.27 ns | -0.54 ns | -0.19 ns | -3.27 ns | -0.15 ** | 0.06 ns | -0.26 ** | 0.23 ns | -0.21 ns | -0.15 ** | 6.42 ns |
| 3 x 6 3,2 | 3.17 ns | -0.21 ns | 1.40 ns | 1.05 ns | 2.41 ns | -0.51 ns | -0.32 ns | 0.01 ns | -0.95 ns | -0.13 * | 0.28 ** | 0.32 ** | -0.15 ns | 0.60 ** | -0.13 * | -7.26 ns |
| 3 x 7 3,4 | 9.84 ns | -0.76 ns | -0.83 ns | -0.51 ns | -0.70 ns | -3.06 * | -0.32 ns | -0.16 ns | -2.68 ns | 0.22 ** | -0.07 ns | -0.45 ** | 0.31 * | -0.52 ** | 0.22 ** | -11.96 ** |
| SCA Var. | 190.2734 | 0.1032 | 0.7275 | 0.777 | 5.6083 | 2.8728 | 0.5028 | 0.045 | 6.5946 | 0.044 | 0.0421 | 0.3236 | 0.3357 | 0.5211 | 0.044 | 173.8821 |

**Supplementary Table S7.** Percentage contributions of lines, testers and their interaction to the total variation under cross the environments.

| Contribution | GP | DG | DS | DF | DBO | PH | NB | BW | YP | PC | ChA | ChB | ChAB | ChT | CTe | CMS |
| --- | --- | --- | --- | --- | --- | --- | --- | --- | --- | --- | --- | --- | --- | --- | --- | --- |
| L | 12.92 | 14.29 | 41.78 | 40.97 | 70.14 | 5.77 | 10.47 | 17.04 | 21.81 | 12.67 | 22.73 | 34.04 | 18.42 | 34.89 | 12.67 | 3.56 |
| T | 13.93 | 32.91 | 14.12 | 24.75 | 14.93 | 18.91 | 32.1 | 27.07 | 34.48 | 27.96 | 22.17 | 29.69 | 19.75 | 25.56 | 27.96 | 67.66 |
| L x T | 73.15 | 52.8 | 44.1 | 34.28 | 14.94 | 75.33 | 57.43 | 55.89 | 43.71 | 59.37 | 55.11 | 36.27 | 61.83 | 39.55 | 59.37 | 28.78 |
|  | DS |  |  |  |  |  |  |  |  |  |  |  |  |  |  |  |
|  | GP | DG | DS | DF | DBO | PH | NB | BW | YP | PC | ChA | ChB | ChAB | ChT | CTe | CMS |
| L | 28.53 | 23.58 | 21.71 | 32.03 | 39.05 | 53.09 | 23.05 | 2.37 | 26.84 | 47.46 | 15.1 | 3.09 | 0.78 | 5.06 | 47.46 | 3.56 |
| T | 15.41 | 28.3 | 46.47 | 40.98 | 38.17 | 23.57 | 19.56 | 71.33 | 26.3 | 13.58 | 20.6 | 26.84 | 28.04 | 24.87 | 13.58 | 67.66 |
| L x T | 56.07 | 48.11 | 31.81 | 27 | 22.78 | 23.35 | 57.39 | 26.3 | 46.85 | 38.96 | 64.3 | 70.06 | 71.18 | 70.08 | 38.96 | 28.78 |

**Supplementary Table S8.** Mean values of lines and testers (Parents) under NS conditions

| Lines | GP | DG | DS | DF | DBO | PH | BN | BW | YP | PC | ChA | ChB | ChAB | ChT | CTe | CMS |
| --- | --- | --- | --- | --- | --- | --- | --- | --- | --- | --- | --- | --- | --- | --- | --- | --- |
| DTV-9 | 63.33 | 4.33 | 37.00 | 56.00 | 87.67 | 77.00 | 13.00 | 3.58 | 46.58 | 0.12 | 21.43 | 17.82 | 1.55 | 27.74 | 25.42 | 68.77 |
| BT-992 | 53.33 | 5.00 | 37.33 | 57.67 | 93.33 | 61.00 | 11.00 | 3.55 | 39.02 | 0.26 | 22.14 | 16.36 | 1.99 | 28.30 | 26.56 | 54.09 |
| MNH-886 | 73.33 | 4.00 | 39.00 | 56.67 | 91.33 | 77.33 | 11.67 | 4.08 | 47.58 | 0.15 | 22.74 | 16.46 | 2.83 | 28.10 | 26.05 | 33.23 |
| Testers |  |  |  |  |  |  |  |  |  |  |  |  |  |  |  |  |
| MNH-988 | 56.67 | 5.00 | 38.33 | 55.67 | 93.00 | 62.00 | 15.33 | 4.76 | 73.19 | 0.30 | 22.41 | 18.04 | 0.84 | 27.65 | 26.70 | 26.88 |
| DTV-3 | 50.00 | 5.33 | 37.33 | 52.33 | 90.67 | 54.00 | 9.67 | 4.73 | 45.79 | 0.20 | 23.08 | 16.32 | 1.58 | 28.41 | 26.40 | 72.70 |
| DTV-10 | 40.00 | 5.33 | 39.00 | 55.00 | 93.33 | 61.67 | 10.67 | 3.42 | 36.51 | 0.14 | 22.92 | 16.52 | 1.34 | 28.55 | 25.44 | 48.59 |
| BT-252 | 53.33 | 6.33 | 38.67 | 56.00 | 92.00 | 55.33 | 10.67 | 3.39 | 36.22 | 0.30 | 19.95 | 15.22 | 4.75 | 26.17 | 26.30 | 64.42 |
| BT-555 | 56.67 | 5.67 | 37.67 | 55.33 | 86.67 | 55.67 | 9.67 | 3.74 | 36.26 | 0.14 | 21.11 | 15.65 | 1.74 | 26.76 | 25.84 | 86.14 |
| BT-666 | 66.67 | 5.67 | 39.33 | 55.33 | 96.33 | 53.00 | 9.33 | 3.72 | 34.76 | 0.34 | 21.44 | 15.81 | 1.88 | 27.25 | 26.34 | 51.18 |
| FH-942 | 30.00 | 6.67 | 41.00 | 60.00 | 95.00 | 82.00 | 10.67 | 2.63 | 27.99 | 0.10 | 22.05 | 17.47 | 0.95 | 29.22 | 26.17 | 54.29 |

**Supplementary Table S9.** Mean values of lines and testers (Parents) under DS conditions

| Lines | GP | DG | DS | DF | DBO | PH | BN | BW | YP | PC | ChA | ChB | ChAB | ChT | CTe | CMS |
| --- | --- | --- | --- | --- | --- | --- | --- | --- | --- | --- | --- | --- | --- | --- | --- | --- |
| DTV-9 | 56.67 | 3.67 | 42.00 | 54.67 | 82.67 | 43.67 | 9.33 | 3.38 | 29.58 | 0.46 | 24.71 | 17.25 | 1.43246 | 29.42 | 26.88 | 68.77 |
| BT-992 | 56.67 | 4.00 | 37.33 | 52.67 | 84.00 | 38.00 | 10.00 | 3.16 | 30.53 | 0.97 | 25.33 | 17.85 | 1.41905 | 30.04 | 27.33 | 54.09 |
| MNH-886 | 73.33 | 5.33 | 40.00 | 57.33 | 84.00 | 40.67 | 9.00 | 3.55 | 31.89 | 0.77 | 24.69 | 17.7 | 1.42316 | 29.44 | 27.72 | 33.23 |
| Testers |  |  |  |  |  |  |  |  |  |  |  |  |  |  |  |  |
| MNH-988 | 53.33 | 5.33 | 42.67 | 61.33 | 91.67 | 35.67 | 8.33 | 2.42 | 19.97 | 0.34 | 25 | 17.12 | 1.46028 | 30.01 | 28.04 | 26.88 |
| DTV-3 | 50.00 | 5.67 | 41.00 | 57.00 | 88.33 | 35.00 | 8.67 | 3.35 | 29.1 | 0.54 | 24.45 | 17.27 | 1.41575 | 29.65 | 27.94 | 72.7 |
| DTV-10 | 40.00 | 5.33 | 40.67 | 58.33 | 89.33 | 43.00 | 8.33 | 2.95 | 24.55 | 0.43 | 24.48 | 16.58 | 1.47648 | 28.84 | 27.87 | 48.59 |
| BT-252 | 56.67 | 6.33 | 40.67 | 55.33 | 89.33 | 34.33 | 9.33 | 3.21 | 29.78 | 0.46 | 21.21 | 15.97 | 1.32812 | 27.23 | 28.96 | 64.42 |
| BT-555 | 50.00 | 5.67 | 40.67 | 56.00 | 85.33 | 36.33 | 7.67 | 3.25 | 24.96 | 0.57 | 23.51 | 16.1 | 1.46025 | 29.49 | 27.41 | 86.14 |
| BT-666 | 63.33 | 6.33 | 41.67 | 58.33 | 94.33 | 33.67 | 7.67 | 2.72 | 20.7 | 0.24 | 23.48 | 17.96 | 1.30735 | 29.8 | 27.58 | 51.18 |
| FH-942 | 30.00 | 6.33 | 45.33 | 59.67 | 95.33 | 41.00 | 7.00 | 2.24 | 15.6 | 0.82 | 24.24 | 17.19 | 1.41012 | 29.38 | 27.99 | 54.29 |

**Supplementary Table S10.** Mean values of 21 hybrids for all studied traits under NS conditions.

| Cross | GP | DG | DS | DF | DBO | PH | BN | BW | YP | PC | ChA | ChB | ChAB | ChT | CTe | CMS |
| --- | --- | --- | --- | --- | --- | --- | --- | --- | --- | --- | --- | --- | --- | --- | --- | --- |
| 1 x 1 | 76.67 | 4.11 | 37.33 | 54.33 | 87.67 | 59.2 | 10.67 | 4.71 | 50.32 | 0.34 | 22.87 | 15.82 | 2.28 | 27.68 | 26.84 | 88.86 |
| 1 x 2 | 63.33 | 4.33 | 36.33 | 54.67 | 86 | 50.33 | 11.33 | 4.51 | 51.19 | 0.41 | 21.89 | 16.06 | 1.79 | 27.95 | 27.91 | 61.14 |
| 1 x 3 | 73.33 | 4.00 | 36.33 | 53.67 | 85.01 | 55.12 | 13.67 | 3.67 | 50.26 | 0.09 | 21.67 | 15.8 | 2.09 | 27.47 | 24.89 | 50.63 |
| 1 x 4 | 56.67 | 3.00 | 35.67 | 51.33 | 82.33 | 55.67 | 11.67 | 3.49 | 40.75 | 0.15 | 21.84 | 15.66 | 2.81 | 27.49 | 25.55 | 13.27 |
| 1 x 5 | 70.01 | 4.33 | 32.33 | 54.67 | 88.33 | 61.01 | 10.67 | 4.07 | 43.32 | 0.34 | 21.56 | 16.09 | 1.42 | 27.65 | 26.34 | 90.51 |
| 1 x 6 | 90.1 | 4.00 | 37.33 | 53.67 | 85.12 | 66 | 9.67 | 2.69 | 26.18 | 0.13 | 19.93 | 15.31 | 3.05 | 26.24 | 26.43 | 61.28 |
| 1 x 7 | 83.33 | 4.00 | 36.33 | 52.67 | 85.67 | 64.67 | 11.67 | 3.92 | 45.72 | 0.14 | 21.53 | 16.16 | 1.29 | 27.69 | 25.74 | 55.6 |
| 2 x 1 | 33.33 | 5.33 | 35.21 | 51.33 | 89.33 | 51.67 | 11.33 | 4.91 | 55.69 | 0.27 | 19.87 | 15.45 | 2.4 | 26.32 | 26.57 | 81.34 |
| 2 x 2 | 63.33 | 4.67 | 39.67 | 56.67 | 92.33 | 62.02 | 15.21 | 4.75 | 71.24 | 0.18 | 21.8 | 16.75 | 1.92 | 28.35 | 26.18 | 56.55 |
| 2 x 3 | 66.67 | 4.33 | 36.1 | 57.01 | 90.1 | 68.67 | 11.33 | 4.12 | 46.7 | 0.38 | 21.76 | 16.99 | 3.12 | 28.35 | 27.68 | 29.23 |
| 2 x 4 | 73.33 | 4.67 | 39 | 55.67 | 91.33 | 58.01 | 11.67 | 4.35 | 50.71 | 0.19 | 21.97 | 16.23 | 1.99 | 28.2 | 26.69 | 37.41 |
| 2 x 5 | 70.21 | 4.33 | 38.33 | 54.33 | 89.33 | 56.67 | 11.67 | 4.38 | 51.2 | 0.34 | 22.51 | 16.27 | 1.67 | 28.38 | 27.14 | 76.91 |
| 2 x 6 | 73.33 | 4.00 | 38.01 | 56.12 | 90 | 50.33 | 11.12 | 4.53 | 49.78 | 0.38 | 22.72 | 16.91 | 1.11 | 29.03 | 27.58 | 44.76 |
| 2 x 7 | 76.67 | 3.67 | 37.67 | 54.67 | 89.3 | 53 | 14.11 | 3.65 | 51.04 | 0.12 | 20.99 | 15.98 | 2.04 | 27.97 | 25.12 | 33.06 |
| 3 x 1 | 66.67 | 5.01 | 38.67 | 57.21 | 94.33 | 62.67 | 15.01 | 4.36 | 65.33 | 0.13 | 21.59 | 15.59 | 2.76 | 27.18 | 25.13 | 70.56 |
| 3 x 2 | 76.67 | 3.67 | 41.33 | 57.67 | 96.2 | 62.33 | 12.1 | 3.64 | 43.74 | 0.26 | 20.96 | 17.82 | 0.93 | 29.07 | 26.76 | 35.93 |
| 3 x 3 | 73.33 | 3.67 | 38.67 | 58.67 | 94.01 | 66 | 12.33 | 3.58 | 44.24 | 0.2 | 23.56 | 17.74 | 0.85 | 29.7 | 26.2 | 61.62 |
| 3 x 4 | 66.67 | 4.00 | 37.33 | 54.33 | 88.33 | 58.02 | 12 | 4.28 | 51.42 | 0.14 | 22.82 | 16.75 | 1.21 | 28.87 | 25.14 | 35.28 |
| 3 x 5 | 50.22 | 5.00 | 40 | 59.02 | 97.33 | 57.33 | 11.67 | 3.72 | 43.48 | 0.14 | 23.12 | 16.02 | 2.08 | 28.14 | 25.14 | 55.71 |
| 3 x 6 | 63.33 | 4.14 | 38.12 | 54.67 | 91.33 | 50.01 | 10.67 | 4.55 | 48.33 | 0.26 | 22.62 | 17.95 | 0.84 | 29.67 | 26.66 | 61.67 |
| 3 x 7 | 36.67 | 4.67 | 38.67 | 56 | 92 | 67.67 | 12.12 | 4.24 | 50.74 | 0.12 | 22.7 | 16.45 | 1.45 | 28.55 | 25.12 | 58.05 |

**Supplementary Table S11.** Mean values of 21 hybrids for all studied traits under DS conditions.

| Cross | GP | DG | DS | DF | DBO | PH | BN | BW | YP | PC | ChA | ChB | ChAB | ChT | CTe | CMS |
| --- | --- | --- | --- | --- | --- | --- | --- | --- | --- | --- | --- | --- | --- | --- | --- | --- |
| 1 x 1 | 70.10 | 4.13 | 41.33 | 57.67 | 87.33 | 39.33 | 7.67 | 3.47 | 26.49 | 0.91 | 24.08 | 15.97 | 1.51 | 27.98 | 28.75 | 88.86 |
| 1 x 2 | 70.14 | 4.33 | 40.00 | 57.00 | 86.67 | 36.00 | 6.01 | 3.55 | 21.40 | 0.99 | 24.00 | 16.60 | 1.45 | 28.71 | 29.20 | 61.14 |
| 1 x 3 | 73.33 | 4.33 | 39.33 | 53.33 | 79.33 | 41.00 | 7.11 | 3.69 | 25.86 | 1.04 | 23.77 | 16.65 | 1.43 | 28.76 | 26.93 | 50.63 |
| 1 x 4 | 53.33 | 3.67 | 38.33 | 54.13 | 78.12 | 43.67 | 6.10 | 3.26 | 19.69 | 1.04 | 24.55 | 16.64 | 1.48 | 28.85 | 27.59 | 13.27 |
| 1 x 5 | 76.67 | 4.33 | 36.67 | 53.33 | 75.67 | 39.67 | 7.67 | 3.49 | 26.67 | 1.01 | 23.52 | 16.65 | 1.41 | 28.62 | 28.35 | 90.51 |
| 1 x 6 | 70.13 | 4.00 | 37.67 | 54.00 | 83.12 | 42.00 | 9.00 | 2.45 | 22.01 | 0.92 | 21.27 | 15.66 | 1.36 | 27.10 | 28.35 | 61.28 |
| 1 x 7 | 93.33 | 3.67 | 43.12 | 57.67 | 84.67 | 45.33 | 8.33 | 3.03 | 25.29 | 0.68 | 23.65 | 16.18 | 1.46 | 28.18 | 27.42 | 55.60 |
| 2 x 1 | 53.33 | 5.33 | 41.00 | 56.67 | 89.33 | 36.67 | 7.67 | 3.57 | 27.31 | 0.74 | 21.90 | 15.66 | 1.40 | 27.59 | 28.31 | 70.56 |
| 2 x 2 | 73.33 | 3.67 | 41.33 | 58.33 | 94.33 | 38.00 | 7.67 | 3.16 | 24.17 | 0.33 | 23.88 | 15.87 | 1.50 | 27.95 | 27.51 | 35.93 |
| 2 x 3 | 63.33 | 4.67 | 40.67 | 56.11 | 82.67 | 35.67 | 8.33 | 3.36 | 28.10 | 0.52 | 23.83 | 16.36 | 1.46 | 28.43 | 29.20 | 61.62 |
| 2 x 4 | 53.33 | 3.67 | 40.67 | 57.10 | 86.33 | 39.33 | 7.67 | 3.76 | 28.87 | 0.66 | 24.05 | 16.14 | 1.49 | 28.22 | 28.35 | 35.28 |
| 2 x 5 | 56.67 | 4.33 | 41.10 | 57.11 | 86.67 | 34.67 | 7.67 | 3.70 | 28.36 | 0.38 | 24.52 | 17.53 | 1.40 | 29.74 | 28.52 | 55.71 |
| 2 x 6 | 53.33 | 4.33 | 40.67 | 57.12 | 82.11 | 37.00 | 7.67 | 3.17 | 24.27 | 0.79 | 24.05 | 16.62 | 1.45 | 28.75 | 29.37 | 61.67 |
| 2 x 7 | 43.33 | 5.67 | 42.67 | 59.33 | 89.67 | 42.67 | 7.67 | 3.02 | 23.16 | 0.25 | 24.29 | 17.70 | 1.37 | 29.91 | 26.37 | 58.05 |
| 3 x 1 | 26.67 | 5.67 | 40.11 | 58.01 | 87.33 | 35.67 | 8.00 | 3.67 | 29.31 | 0.74 | 23.88 | 16.81 | 1.42 | 28.90 | 26.87 | 81.34 |
| 3 x 2 | 40.14 | 5.12 | 41.67 | 56.67 | 91.00 | 35.00 | 10.00 | 3.48 | 34.83 | 0.98 | 23.12 | 16.25 | 1.42 | 28.41 | 28.74 | 56.55 |
| 3 x 3 | 46.67 | 5.00 | 39.67 | 54.67 | 85.10 | 36.67 | 8.33 | 3.94 | 32.85 | 0.75 | 23.70 | 15.75 | 1.50 | 27.57 | 27.95 | 29.23 |
| 3 x 4 | 73.33 | 4.33 | 40.00 | 57.33 | 87.67 | 34.67 | 7.67 | 3.54 | 27.19 | 0.23 | 25.38 | 16.53 | 1.54 | 28.79 | 26.37 | 37.41 |
| 3 x 5 | 66.67 | 5.13 | 40.00 | 59.11 | 89.10 | 35.00 | 7.67 | 3.37 | 25.84 | 0.35 | 25.20 | 16.65 | 1.51 | 28.93 | 26.49 | 76.91 |
| 3 x 6 | 56.67 | 4.33 | 41.67 | 58.33 | 90.11 | 34.67 | 8.67 | 2.88 | 24.95 | 0.54 | 24.89 | 16.56 | 1.50 | 28.83 | 28.20 | 44.76 |
| 3 x 7 | 73.33 | 4.15 | 42.10 | 59.11 | 90.01 | 35.33 | 8.33 | 2.84 | 23.43 | 0.68 | 24.11 | 16.22 | 1.49 | 28.33 | 26.80 | 33.06 |

**Supplementary Table S12.** Area, Production and Yield of Cotton

| **Year** | **Area** | | **Production** | | **Yield** | |
| --- | --- | --- | --- | --- | --- | --- |
|  | Hectares | %Change | Bales | %Change | Kgs/Hec | %Change |
| 2018-19(P) | 2,373 | -12.1 | 9,861 | -17.5 | 707 | -6.1 |
| 2017-18 | 2,700 | 8.5 | 11,946 | 11.9 | 753 | 3.1 |
| 2016-17 | 2,489 | -14.2 | 10,671 | 7.6 | 730 | 25.3 |
| 2015-16 | 2,902 | -2.0 | 9,917 | -29.0 | 582 | -27.4 |
| 2014-15 | 2,961 | - | 13,960 | - | 802 | - |

P: Provisional (July-March) Source: Pakistan Bureau of Statistics

**Supplementary Figure S1.** Cultivation area (000 hectare), Cotton production (000bales), and Yield (kgs/hec)


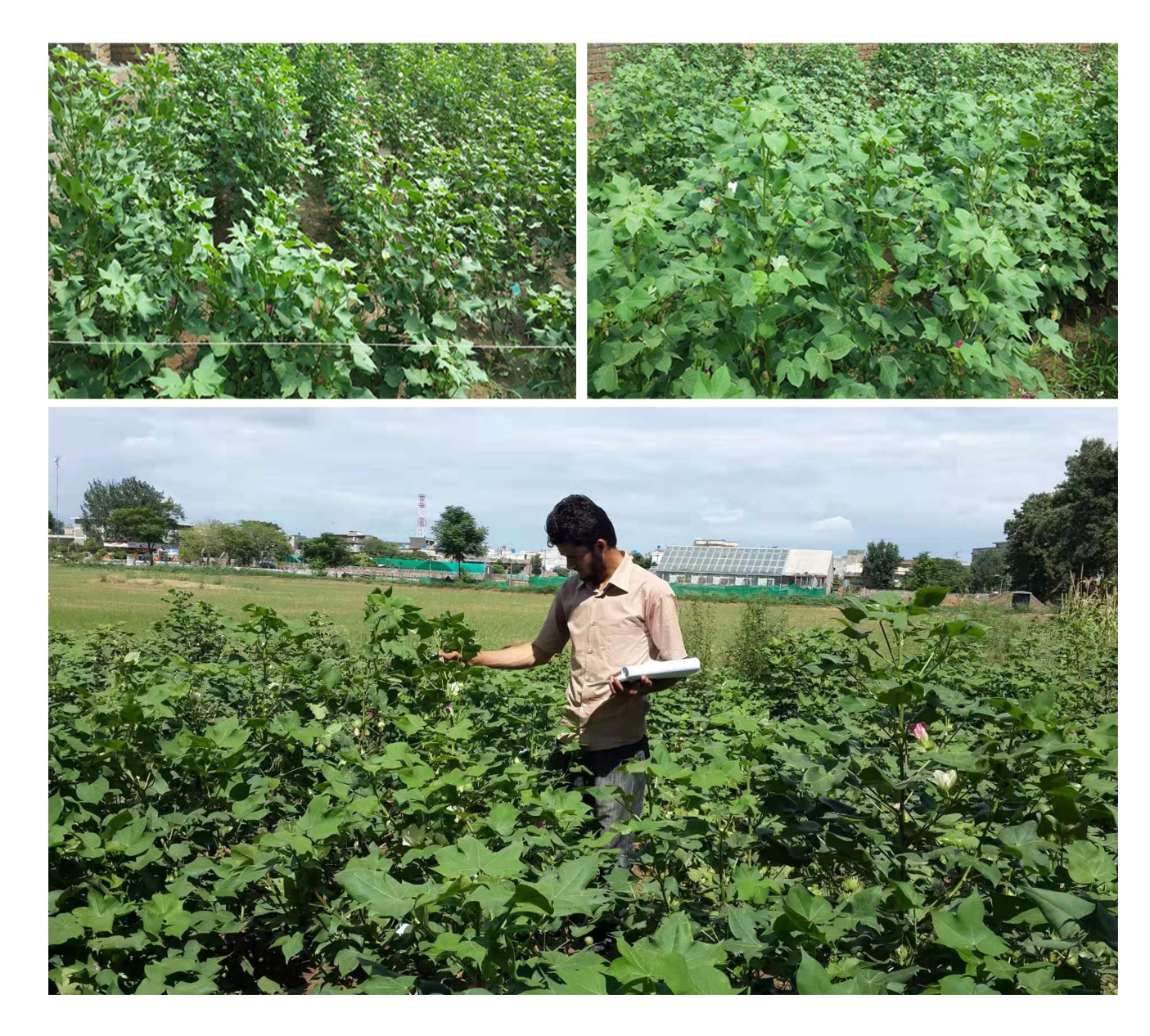


**Supplementary Figure S2.** Field performance and growth of cotton in Rawalpindi district, Pakistan
